# In vitro assays for investigating the immunomodulatory properties of human mesenchymal stromal cells

**DOI:** 10.1186/s13287-026-04920-x

**Published:** 2026-02-07

**Authors:** Laura Lykke Lethager, Stine Bangsgaard, Ellen Mønsted Johansen, Abbas Ali Qayyum, Jan Pravsgaard Christensen, Annette Ekblond, Morten Juhl Nørgaard, Lisbeth Drozd Højgaard

**Affiliations:** 1 Cardiology Stem Cell Centre, The Heart Centre, Copenhagen University Hospital (Rigshospitalet), Blegdamsvej 9, 2100 Copenhagen, Denmark; 2 Department of Cardiology, Hvidovre Hospital, Hvidovre, Denmark; 3 Department of Clinical Medicine, University of Copenhagen, Copenhagen, Denmark; 4 Department of Immunology and Microbiology, Faculty of Health and Medical Sciences, University of Copenhagen, Copenhagen, Denmark; 5 Cell to Cure Aps, Birkerød, Denmark

**Keywords:** Cell therapy, Bioassay, In vitro assay, Mesenchymal stromal cell, Stem cell, Immunomodulation, Analytical methods, Immune cells, Methods, Investigative techniques

## Abstract

Mesenchymal stromal cells (MSCs) are widely recognized for their immunomodulatory properties, which underpin their therapeutic potential in inflammatory and immune-mediated diseases. Although MSC therapies have consistently proven safe, clinical efficacy remains inconclusive, maybe due to incomplete understanding of MSC interactions with the immune environment. This review evaluates current trends in MSC immunomodulation research, based on 318 studies published since 2019 until medio 2024. The most frequently used assays included characterization, proliferation, and polarization, employing methods such as flow cytometry, enzyme-linked immunosorbent assays and colorimetric assays, and polymerase chain reaction. Many studies incorporated strategies for priming of MSCs or included immune cells, most commonly peripheral blood mononuclear cells, T cells, and macrophages. We identify key sources of variability and propose a minimum reporting checklist including MSC source, priming conditions, assay design, and immune cell characteristics. We further recommend implementation of multi-assay workflows combining phenotypic characterization with at least one functional assay. These measures may improve transparency, comparability across studies, and guide robust assay design.

**Graphical Abstract:**

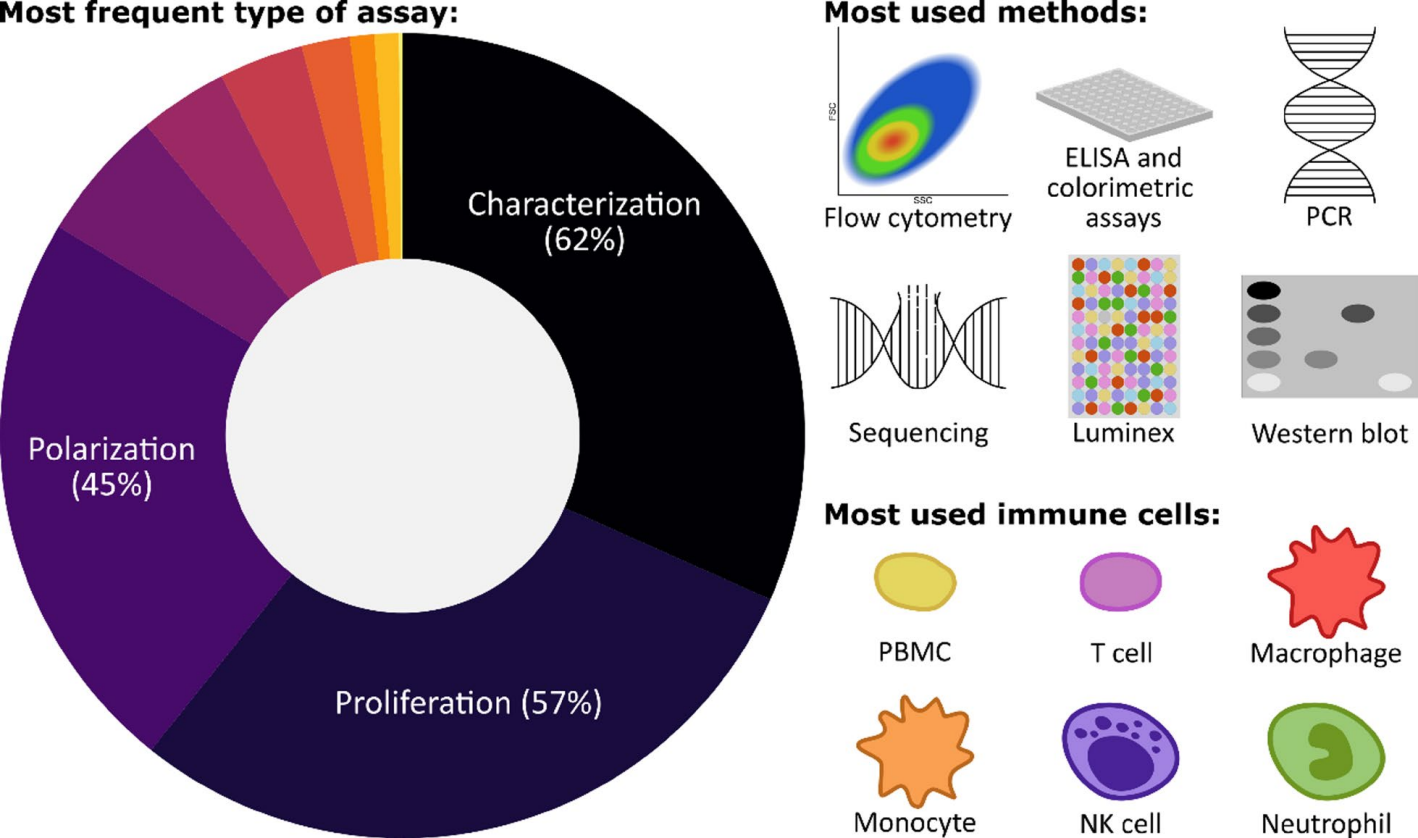

**Supplementary Information:**

The online version contains supplementary material available at 10.1186/s13287-026-04920-x.

## Background

Mesenchymal stromal cells (MSCs) were previously coveted for their role in tissue homeostasis and maintenance of the stem cell niche, because of their ability to differentiate into various cell types. Furthermore, an important biological property was thought to be their ability to sense and home to damaged tissue in the local environment. However, in recent years, the focus has shifted, and now the primary role of MSCs is thought to be as orchestrators of the immune response [1, 2]. Inflammation is a complex state characterized by interplay between pro- and anti-inflammatory cytokines that regulate activity of leucocytes and surrounding tissue and plays a vital role in many acute and chronic progressive diseases. Therefore, the ability to adequately regulate the immune response is crucial in disease development and progression, and as MSCs have both immunomodulatory and anti-inflammatory properties, there is increasing interest in exploring their therapeutic potential [3–10]. MSCs affect their environment through paracrine signalling and crosstalk with innate and adaptive immune cells, affecting numerous biological processes, e.g., enabling them to alter proliferation rate and polarization state. Thereby, they assist in maintaining homeostasis of the tissue environment [4, 11, 12]. Paracrine signalling is facilitated by the MSC secretome, which consists of soluble factors such as cytokines, chemokines, or growth factors as well as extracellular vesicles and apoptotic bodies [13, 14]. MSCs are highly dynamic and plastic cells that perform different functions depending on the environment to which they are exposed. They can trigger a response from the immune system in response to external stimuli, such as damage or infection, yet they can also respond to immune activation by becoming immunosuppressive, thereby preventing an excessive immune response [7, 11]. However, the exact mechanisms by which these functions are exerted remain unknown. Owing to their immunomodulatory properties, MSCs have been suggested as a novel therapy for numerous clinical indications [9, 11, 15–28]. Despite the large number of clinical trials in which MSC therapy has been proven safe, the results concerning its efficacy have been inconclusive, maybe due to the complexity of the interactions between the tissue environment of patients and MSCs, which are not yet fully understood [7, 17–19, 29]. Various assays have been used to explore different aspects of MSC characteristics, but currently, there is no consensus for the existing bioassays on either design nor endpoints and thereby variability is introduced. As such, the development of relevant assays following minimum reporting standards is crucial for advancing the understanding of MSCs in an immunological context. This review aims to evaluate current trends in types of assays used to assess the MSC immunomodulatory potential, identify key challenges, and serve as a guide for future research.

## Methods

### Inclusion criteria

**Fig. 1:**
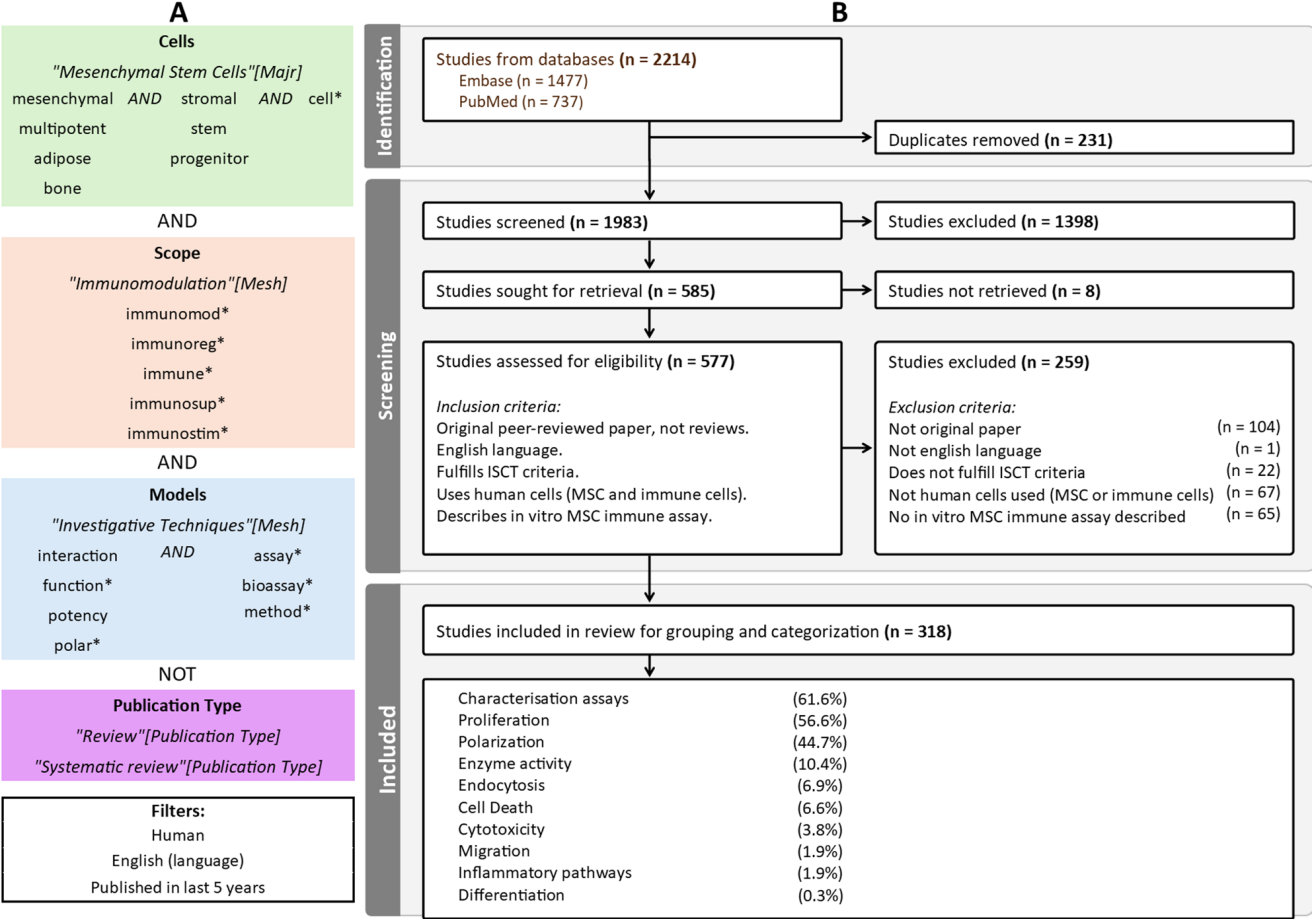
Search strategy and PRISMA diagram. **A** Search string used to include studies. Both medicinal subject headings (Mesh) (in cursive) and title-abstract terms are included. Reviews and systematic reviews were excluded. Filtered to only include studies on human cells written in English in the 5 years prior to conducting the search (15aug2024). **B** PRISMA diagram illustrating the screening process. The search was conducted in both Embase and PubMed (Medline). First, duplicates were removed before the initial screening of the title and abstract by one reviewer. The full texts of the included articles were screened by two reviewers, and a third reviewer resolved conflicts, omitting those with no online access. Studies were included or excluded during full-text screening based on the listed inclusion or exclusion criteria. In total, 318 studies were included. The criteria for MSCs were as defined by the International Society for Cellular Therapy (ISCT) [30]. The categorization into types of assays was performed by the reviewers. The percentages reflect the distribution of type of assay in relation to the number of included studies

The search string used medicinal subject headings and title-abstract terms to include papers on MSCs, immunomodulation, and bioassays or methods (Fig. 1A). The search was conducted on the 15th of August 2024 in both the Embase and PubMed (Medline) databases, followed by screening of titles and abstracts and subsequent full-text screening in Covidence, as shown in Fig. 1B.

**Fig. 2:**
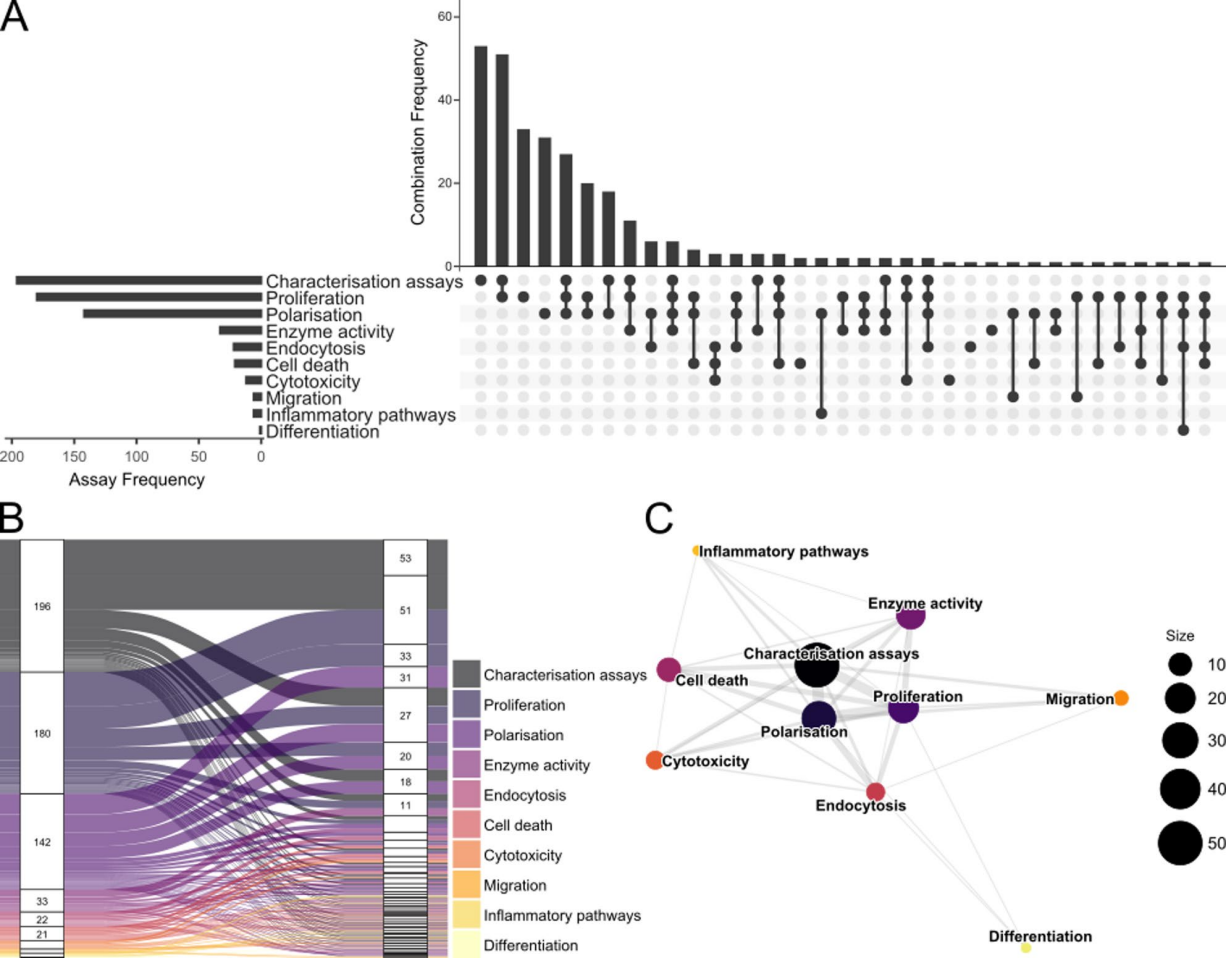
Overview of assay usage across the included studies. **A** Assay frequency and co-occurrence. UpSet plot showing which assay types are most commonly used (left) and how frequently specific combinations occur (top). The matrix highlights the composition of each combination. **B** Distribution of studies across combinations. Box heights indicate how many studies contain each assay combination, providing an overview of how heterogeneously studies cluster across the field. **C** Assay co-usage relationships. Network representation illustrating how assay types relate to each other based on co-usage patterns. Node size reflects overall frequency, and edge thickness indicates how often two assay types appear together

In brief, the included papers had to be original, peer-reviewed, written in English, and published since 2019 and until the search was conducted on 15aug2024. Furthermore, the MSCs must conform to the minimal criteria described by the International Society for Cellular Therapy (ISCT) [30], and both the MSCs and other included cells should be of human origin. The papers should describe an in vitro assay investigating the immunomodulatory functions of MSCs. In total, 318 papers were included based on the established inclusion criteria (Fig. 1B). Further analysis of the included assays was conducted using R extension for Visual Studio Code (version 2.8.6) in Visual Studio Code (version 1.103.1), employing packages readxl [31], tidyverse [32], openxlsx [33], viridis [34], igraph [35], intergraph [36], sna [37], GGally [38], ggalluvial [38], and UpSet [39]. The assays were divided into ten predefined categories depending on the investigated immunomodulatory effect of the MSCs (Fig. 1B). A complete overview can be seen in Table 1, and an overview of assay types used across the included studies is shown in Fig. 2. Most papers described multiple assays and/or used several analytical methods to illustrate the multifaceted nature of MSC function, and a mean of 2.7 assays per paper were included. Both alone and in combination with other assays, the most used assays where characterization, proliferation, and polarisation, while assays investigating cell death, inflammatory pathways, migration, and differentiation were only used in combination with other assays (Fig. 2).

**Table 1:** Overview of the types of assays and methods used in the included assays

Type of assay	Included immune cells	Method	References
Characterization assays (61.6%)	No	PCR (22.8%)	[5, 8, 12, 16, 51, 52, 55, 62, 73, 75, 76, 78, 85, 89, 92, 102, 105, 112, 115, 118, 121, 123–129, 133, 157, 166, 170, 174, 175, 179, 181, 182, 186, 198, 214, 216, 217, 226, 227, 238–241, 243–245, 275–285][342-344]
		ELISA (16.5%)	[13, 52, 58, 63, 69, 74, 76, 88, 99, 107, 112, 121, 125, 128, 138, 143–[145, 159, 165, 166, 168, 181, 184, 196, 217, 222, 226–228, 231, 239–241, 243, 245, 247, 248, 251, 265, 268, 278, 279, 282–284, 286–294]
		Sequencing (13.2%)	[6, 10, 19, 55, 66, 69, 100, 107, 120, 127, 134–141, 147, 155, 161, 171, 172, 179, 181, 196–198, 217, 222, 236, 251, 269, 276, 279, 281, 283, 285, 290, 293, 295–297]
		Western blot (7.8%)	[15, 16, 62, 73, 74, 78, 92, 102, 115, 121, 127, 128, 133, 147, 157, 164, 171, 182, 186, 190, 226, 244, 245, 292, 298, 299]
		Luminex (7.2%)	[55, 57, 58, 106, 136, 138, 148–151, 164, 167, 180, 223, 226, 229, 236, 246, 276, 279, 289, 299–301]
		Flow cytometry (6.9%)	[12, 19, 66, 71, 77, 142, 152, 157, 159, 180, 182, 184, 221, 234, 238, 239, 247, 251, 282, 290, 302, 303]
		Mass spectrometry (3.3%)	[58, 87, 100, 109, 122, 130, 136, 148, 246, 250, 277]
		Colorimetric assay (2.7%)	[146, 162, 190, 228, 239, 241, 244, 248, 292]
		Microscopy (1.2%)	[102, 121, 128, 288]
		NMR spectroscopy (0.6%)	[246, 250]
	PBMC, Macrophages, T cells, Monocytes	ELISA (7.2%)	[45, 53, 68, 81, 89, 91, 95, 97, 109, 154–158, 174, 178, 192, 193, 201, 202, 208, 209, 225, 245]
	PBMC, Macrophages, T cells	PCR (5.1%)	[80– [83, 105, 109, 154, 156, 158–161, 176, 177, 193, 206, 304]
	PBMC, T cells, Monocytes, Glial cells	Luminex (1.8%)	[5, 42, 83, 173, 305, 306]
	PBMC, Macrophages	Flow cytometry (0.9%)	[154, 199, 210]
	PBMC, T cells	Sequencing (0.9%)	[156, 206, 207]
	T cells, PBMC	Western blot (0.9%)	[119, 210, 305]
	PBMC, T cells	Colorimetric assay (0.3%)	[85]
	Monocytes	Mass spectrometry (0.3%)	[209]
	PBMC	Microscopy (0.3%)	[210]
Proliferation (56.6%)	PBMC, T cells, B cells, NK cells	Flow cytometry (71.3%)	[3–5, 9, 13, 14, 17, 18, 29, 41–43, 45, 58, 62, 66, 71, 73, 77, 78, 81, 82, 85, 99, 114, 117, 120–122, 127, 131–135, 138, 139, 147, 149, 156, 157, 159, 160, 162, 163, 165, 167, 169–171, 175, 179, 182, 184–186, 190, 193, 194, 196, 197, 200, 203, 206, 207, 211, 214, 217, 218, 220, 221, 225, 227, 232–237, 239–242, 244–251, 253, 256, 257, 261, 263, 264, 267, 268, 271, 276–279, 282, 286, 289, 290, 294–296, 298, 302, 304, 307–324]
	PBMC, T cells, Macrophages	Colorimetric assay (17%)	[15, 16, 50, 59, 68, 73, 79, 83, 94, 99, 110, 119, 126, 142, 166, 168, 170, 198, 199, 201, 204, 205, 220, 231, 232, 251, 269, 284, 293, 325, 326]
	PBMC, T cells	Radiation-based assay (6.4%)	[69, 72, 107, 140, 141, 164, 255, 270, 299, 327–329]
	PBMC, T cells	ELISA (3.7%)	[70, 103, 140, 195, 219, 288, 330]
	Macrophages	Cell count (0.5%)	[154]
	PBMC	Live Cell Imaging (0.5%)	[188]
	Macrophages	Microscopy (0.5%)	[98]
Polarization (44.7%)	PBMC, T cells, Macrophages, Dendritic cells, Monocytes, B cells, Full blood	Flow cytometry (44.7%)	[3, 7, 11, 13, 14, 43, 44, 47–49, 53, 56, 58, 60, 64, 70, 78–81, 85, 88, 90, 97–99, 102–105, 112, 113, 122, 130, 132, 134, 140, 146, 151, 160–164, 169, 170, 174–178, 183, 187, 189, 191, 192, 201, 207, 211, 212, 218, 220, 225, 230, 237, 247, 254, 255, 259, 260, 266, 276, 279, 286, 290, 298, 301, 309, 316, 317, 319, 331–336]
	PBMC, Macrophages, T cells, B cells, NK cells, Dendritic cells, Monocytes	ELISA (25.9%)	[3, 13, 16, 19, 44, 58, 59, 64, 65, 70, 79, 80, 84, 90, 91, 94, 99, 100, 104, 105, 107, 108, 110, 111, 115–117, 120, 133, 147, 151, 178, 185, 199, 200, 207, 212, 218, 240, 249, 254, 259, 263, 268, 308, 322, 327, 335, 337–339]
	Macrophages, PBMC, T cells, Monocytes, NK cells, B cells	PCR (18.3%)	[19, 44, 46, 48, 61, 64, 65, 67, 68, 84–86, 89, 90, 92, 94, 100, 102–104, 110, 113, 114, 116, 118, 178, 187, 199, 218, 237, 239, 254, 266, 304, 325, 340]
	Macrophages, T cells, PBMC, Dendritic cells, B cells, Full blood	Luminex (6.6%)	[82, 87, 93, 101, 139, 206, 207, 257, 261, 319, 321, 331, 338]
	Macrophages, PBMC	Microscopy (1.5%)	[101, 325, 335]
	T cells	Colorimetric assay (1%)	[121, 341]
	Macrophages, T cells	Western blot (1%)	[335, 341]
	Macrophages, Monocytes	OLINK (0.5%)	[191]
	NK cells	Sequencing (0.5%)	[117]
Enzyme activity (10.4%)	No	Colorimetric assay (68.6%)	[15, 16, 63, 67, 70, 77, 100, 114, 168, 187, 203, 223, 238–248, 250]
		Mass spectrometry (5.7%)	[251, 252]
		Western blot (5.7%)	[231, 252]
	PBMC, T cells	Colorimetric assay (14.3%)	[45, 77, 80, 159, 249]
	T cells, NK cells	Mass spectrometry (5.7%)	[207, 253]
Endocytosis (6.9%)	Macrophages, PBMC, Neutrophils, Dendritic cells, Full blood, Monocytes, T cells	Flow cytometry (52.2%)	[13, 14, 87, 90, 113, 133, 185, 191, 249, 254–256]
	Macrophages, Neutrophils, T cells, PBMC	Microscopy (43.5%)	[96, 97, 103, 109, 133, 213, 215, 257–259]
	Macrophages	Live Cell Imaging (4.3%)	[260]
Cell death (6.6%)	No	Microscopy (4%)	[115]
		PCR (4%)	[54]
		Western blot (4%)	[54]
	PBMC, Neutrophils, Macrophages, T cells, B cells, NK cells	Flow cytometry (64%)	[46, 60, 99, 127, 140, 169, 185, 213, 215, 237, 245, 253, 258, 261–263]
	PBMC	Cell count (4%)	[74]
	T cells	Colorimetric assay (4%)	[121]
	PBMC	ELISA (4%)	[264]
	T cells	Microscopy (4%)	[261]
	T cells	PCR (4%)	[261]
	T cells	Western blot (4%)	[261]
Cytotoxicity (3.8%)	Neutrophils, NK cells, PBMC	Colorimetric assay (40%)	[118–120, 213, 215, 258]
	PBMC, T cells, NK cells	Flow cytometry (33.3%)	[40, 118, 120, 220, 227]
	PBMC	Cell count (6.7%)	[231]
	PBMC	Live Cell Imaging (6.7%)	[265]
	Neutrophils	Microscopy (6.7%)	[97]
	NK cells	Radiation-based assay (6.7%)	[40]
Migration (1.9%)	PBMC, Macrophages	Western blot (50%)	[88, 106]
	Monocytes, PBMC	Flow cytometry (18.2%)	[11, 58]
	Macrophages, Monocytes, PBMC	PCR (18.2%)	[11, 17]
	Macrophages, Monocytes	ELISA (9.1%)	[11]
	Neutrophils	Live Cell Imaging (9.1%)	[87]
	PBMC	Luminex (9.1%)	[17]
	Macrophages, Monocytes	Sequencing (9.1%)	[11]
	Macrophages, Monocytes	Western blot (9.1%)	[11]
Inflammatory pathways (1.9%)	No	Western blot (33.3%)	[170, 243]
		PCR (16.7%)	[152]
	PBMC, Macrophages	Western blot (50%)	[46, 93, 266]
Differentiation (0.3%)	Dendritic cells, Macrophages	PCR (100%)	[255]

The type of assay appears in order of most to least frequently described. Percentage of each type of assay is calculated based on total number of papers included. Assays not including immune cells are indicated by “No”, and the immune cells included are listed by frequency. Methods are listed for each type of assay from most to least frequently used

### Variation in MSC sources

**Fig. 3:**
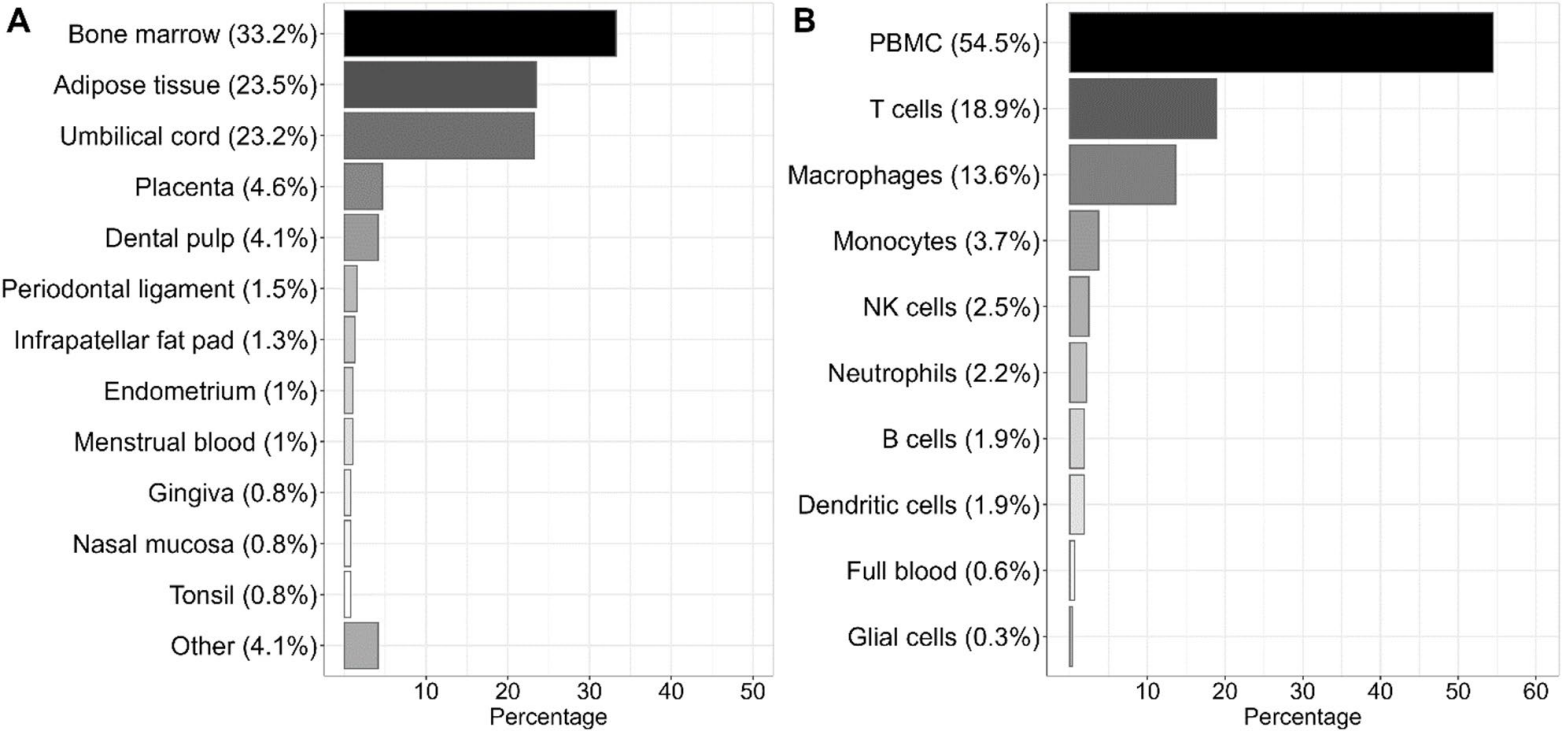
Overview of MSC tissue sources and immune cells. **A** Chart illustrating the tissue origin of MSCs across all included papers. MSCs were isolated from 28 different tissue sites. The “Other” category includes MSCs of tissue origins, which are described in only one paper. **B** Use of immune cells in coculture assays in the included papers. The chart does not discern between use of primary cells or cell lines. PBMC: Peripheral blood mononuclear cells, NK: Natural killer

This review included papers that used MSCs isolated from 28 different tissues, the most common of which were bone marrow, adipose tissue, and the umbilical cord (Fig. 3A).

These sources accounted for 80% of the included MSCs. While most studies used MSCs from healthy donors, cells were also isolated from patients with inflammatory diseases [7, 40–67] or diabetes [68, 69], or from isolated tumor tissue [70, 71]. In a few cases, MSCs were isolated from deceased donors [29, 72], and one paper used immortalized cell lines [73]. Comparative studies revealed that the immunomodulatory properties of MSCs can vary considerably, reflecting differences in tissue source [6, 16, 74–78], donor health status (e.g., healthy vs. inflammatory conditions) [57, 70], and donor age [79]. This wide heterogeneity in MSC origin represents a major challenge for interpreting and comparing results across studies, as differences in cell source may significantly influence functional outcomes [70, 77] and similarly, differences in the expression of immunosuppressive cytokines by MSC from different sources also contributes to assay results [76].

### Interactions between MSCs and the immune system

Immune cells can be incorporated to create a proinflammatory environment, enabling inference of MSC modes of action in an immunological setting that closely resembles in vivo settings (see Supplementary Table 1). The most prevalently utilized immune cells were peripheral blood mononuclear cells (PBMCs), isolated T cells, and macrophages (Fig. 3B). PBMCs are a heterogeneous cell population containing lymphoid cells such as T cells, B cells, monocytes, and natural killer (NK) cells, hence representing a diverse immunological environment with which the MSC may interact [80–83]. They are easy and accessible tools relevant for studying various areas, e.g., the effects of MSCs on the imbalance between T helper (Th)1, Th2 and regulatory T cells (Tregs), which are present in many diseases [44, 64, 84–86]. Some studies used whole blood from healthy donors [87, 88], thus including all blood components. The results obtained from studies using heterogeneous cell populations can be complex to interpret. Hence, it may be more feasible to use isolated cells for investigating the immunomodulatory effects of MSCs, e.g., monocytes that can be differentiated into macrophages known to have specific immunological properties based on their polarization state or isolated T cells [44, 64, 84–86, 89–92]. However, physiological conditions such as inflammation are characterized by cellular interactions and may be better reflected by more complex culture systems. This reflects that researchers face a wide range of possible cell types that can be included depending on the objective and desired outcome of the assay. While most assays used primary immune cells, some relied on cell lines such as THP1 to mimic the functions of monocytes and macrophages [13, 14, 16, 67, 78, 91–116], NK92 cells for NK cells [117–120] and Jurkat cells for T cells [73, 94, 121, 122]. The advantages of cell lines include an unlimited supply of cells, which ensures consistency, ease of use, and reduced interexperimental variation, but their results may not always translate to primary cells. It is therefore recommended that findings be validated using primary cells [93, 107, 117].

## Characterization assays

To understand the immunomodulatory properties of MSCs, their expression of genes and proteins can be characterized to elucidate both constitutive expression and how it is affected by priming or other treatments.

### The immunomodulatory effects of untreated MSCs

MSCs are frequently associated with anti-inflammatory functions, as evidenced via the use of inflammatory gene and protein panels, which show constitutive expression of immunomodulatory markers. The expression of genes in untreated MSCs was analysed via reverse transcription quantitative PCR (qPCR) [51, 52, 121, 123–133] and sequencing [6, 107, 127, 134–141]. The protein contents of the untreated MSC secretome were determined by enzyme-linked immunosorbent assay (ELISA) [52, 74, 107, 121, 125, 138, 142–145] and other colorimetric assays [146], western blotting [74, 121, 127, 133, 147], Luminex [106, 136, 138, 148–151], mass spectrometry [122, 130, 136, 148], transmission electron microscopy [121], and flow cytometry [152]. It is important to explore the inherent expression patterns of MSCs to observe any changes or responses to other stimuli, such as priming, the presence of immune cells, a combination of these, or other types of treatment.

### The immunomodulatory functions of primed MSCs

MSCs can be primed with biological or chemical factors with the purpose of enhancing or suppressing their immunological function (as reviewed in depth by Hezam, Fu [153]). In half of the included assays, the MSCs were primed, especially in assays with MSCs in monoculture. Moreover, the MSCs were primed in fewer than half of the assays that introduced immune cells, which included macrophages [95, 97, 105, 109, 154], PBMCs [5, 45, 85, 155–158], and T cells [45, 85, 159–161]. Since activated immune cells can enhance the immunomodulatory function of MSCs, the amplified effect achieved by priming may not be necessary in assays that already include activated immune cells, as these cells provide the necessary signals to enhance the MSCs regulatory capabilities. MSCs were mainly primed with proinflammatory cytokines such as interferon (IFN)-γ, tumor necrosis factor (TNF)-α, and interleukin (IL)-1β either alone or in combination. Other stimulants, including proinflammatory cytokines and toll-like receptor agonists, were used to explore the immunological functions of MSCs. An overview of the references that used each priming reagent is shown in Supplementary Table 2. By using priming to block or neutralize potential mediators of the immunomodulatory function of MSCs, their involvement and impact on the effect of MSCs can be confirmed [45, 54, 140, 162–165]. A range of priming reagents were used to investigate specific aims; for example, MSC immunomodulation was enhanced with hydrocortisone [166, 167] or dexamethasone [167–169] and inhibited with immune inhibitors such as infliximab [158], tacrolimus, or cyclosporin [72, 167], among others [157, 170, 171]. The methods used to quantify gene and protein expression levels after MSC priming included PCR and sequencing for gene expression and ELISA, flow cytometry, Luminex, western blotting, mass spectrometry, immunofluorescence microscopy, and nuclear magnetic resonance spectroscopy for protein analysis. A complete list of references supporting each method is shown in Supplementary Table 3.

### The immunomodulatory function of MSCs treated with other components

The MSCs were treated with other substances to investigate the effects of other conditions on MSC functionality. These treatments were not always known as immunoregulators or –suppressors and were therefore not necessarily linked to the immunomodulatory properties of MSCs. Nonetheless, their impact on MSC immunomodulatory properties was still assessed. For example, MSCs were treated with blood, plasma, or serum samples from patients [113, 172–177]; antioxidants such as vitamin E and selenium [178]; or environmental factors such as cigarette smoke extract [42], diesel exhaust particles [179], or insecticides [180] to investigate the effects of smoking, air pollution, and pesticide exposure, respectively. Furthermore, the MSCs were transfected to overexpress or knockout specific functions to elucidate their importance in the immunological functions of MSCs [11, 17, 53, 54, 90, 92, 109, 115, 120, 179–198], or they were treated with mitomycin [50, 68, 78, 104, 162, 199–203] or exposed to gamma-irradiation [86, 204, 205] to prevent cell division.

#### Effect of immune cell presence on untreated MSCs

In some cases, the MSCs were left untreated prior to coculture with immune cells to explore the effect of immune cells on the expression of inflammatory markers by the MSCs. This ability was measured by PCR [80–83, 206], sequencing [206, 207], ELISA [81, 91, 208, 209], Luminex [83], mass spectrometry [209], western blot [119, 210], flow cytometry [210], and immunofluorescence microscopy [210]. Both PBMCs [80–83, 119, 206, 208, 210], T cells [207], monocytes [209], and macrophages [91] were used to create a proinflammatory milieu that increased the immunomodulatory potential of MSCs.

#### Effect of experimental conditions on MSCs

During cell culture, numerous factors contribute to the immunological effect of MSCs. In most assays, cells were seeded together directly, enabling both juxtracrine and paracrine interactions. Additionally, the paracrine effects of supernatants harvested from MSC cultures or extracellular vesicles isolated from MSCs were investigated. The effects of MSCs may vary depending on whether they are investigated by direct cell contact, conditioned medium, or extracellular vesicles, as some functions rely on specific cellular interactions [211–215]. The remaining assays used other methods for cell culture, such as three-dimensional cultures [85, 101, 114, 126, 138, 151, 208, 216–218], engineered exofucosylated [219] or lyophilized MSCs [220], gels [91, 106, 125, 154, 198, 201, 209, 216, 221], scaffolds [91, 201, 222, 223], cell sheets [57, 145, 224, 225], or incubation in hypoxic environments [19, 57, 217, 226–230]. Additional experimental conditions included treating immune cells with apoptotic bodies derived from MSCs [14]. The immunomodulatory profile of the MSCs was affected by these experimental conditions, such as the constituents of the growth medium [149, 203], the number of passages [100], or differences in glucose [231] or oxygen [228] levels. The common goal was to create an environment that mimicked the in vivo environment in patients to understand how cell survival and retention after grafting of cells can be improved and ultimately increase the therapeutic potential.

## Proliferation

Proliferation assays assessed the effect of MSCs on the proliferation of various immune cell types, including PBMCs, T cells, macrophages, B cells, and NK cells. To stimulate the proliferation of immune cells, mitogens or activating beads were commonly applied, or immune cells from several donors were used to initiate mixed lymphocyte reactions (MLRs) [3, 18, 156, 157, 196, 202–204, 220, 232–236]. Considerations for the assay design have previously been detailed [4]. In some assays, the effects of the presence of MSCs on the number of live macrophages [98, 154] or macrophage viability [99] were investigated. The proliferation response was measured by flow cytometry, colorimetric assays (including ELISA), radiation-based assays, enzyme-linked immunospot, live-cell imaging, immunofluorescence microscopy, or manual cell counting. A comprehensive mapping of methods, immune cell types, and references is provided in Table 1. In most studies, MSCs suppressed immune cell proliferation, although some studies reported increased proliferation depending on the context: Some assays investigated the proliferation of specific immune cell subsets that increased proliferation after treatment with MSCs, e.g., Tregs [14, 162, 170, 220] or regulatory B cells [163, 237]. Specific conditions, such as high- or low-glucose conditions [231], environmental factors [179], or the blocking of immunomodulatory proteins [45], affected the function of MSCs, resulting in increased proliferation of immune cells, i.e., decreased immunosuppressive effects. MSC-mediated inhibition of immune cell proliferation is extensively used to demonstrate the immunosuppressive effect of MSCs. By including multiple markers, predominantly active immune cell subgroups can be identified. This is important when investigating the modes and mechanisms of action behind the immunomodulatory potential of MSCs and enables the determination of whether reduced proliferation is due to immunosuppression or caused by immune cell killing by MSCs.

## Polarization

Polarization assays investigated the effect of MSCs on phenotypes of various immune cells, primarily PBMCs, macrophages, and T cells, but also monocytes, dendritic cells, B cells, NK cells, and whole blood. Immune cells were exposed to inflammatory stimuli, and macrophages were typically polarized toward a proinflammatory phenotype. The immunomodulatory effects of MSCs were assessed by measuring cell surface and intracellular markers of immune activation or suppression via techniques such as PCR, sequencing, ELISA, flow cytometry, Luminex, western blotting, microscopy, and targeted proteomics (OLINK). A comprehensive list of references associated with each cell type and analytical method is provided in Table 1. The large number of polarization assays reflects the current hypothesis that MSCs interact with resident immune cells and orchestrate their response, thus modulating and resolving the inflammatory response. In the presence of immune cells, MSCs develop a more immunosuppressive phenotype, which was accompanied by activation of immune cells, maturation of dendritic cells, and polarization of macrophages towards an anti-inflammatory phenotype. The phenotype of immune cells can be determined based on surrogate markers, making this type of assay relatively simple. However, the relevance and reliability of any surrogate marker must be thoroughly established.

## Enzyme activity

Enzyme activity assays primarily measured the activity of indoleamine 2,3-dioxygenase (IDO), which is part of tryptophan metabolism. IDO activity was measured by colorimetric assays [15, 16, 45, 63, 67, 70, 77, 80, 100, 114, 159, 168, 187, 203, 223, 238–250] and high-performance liquid chromatography [251]. MSCs do not express IDO constitutively, but IDO expression is upregulated by proinflammatory stimuli; therefore, the MSCs were primed [15, 16, 63, 67, 70, 77, 100, 114, 168, 187, 203, 223, 238–248, 250–252], cocultured with PBMCs [80], or both [45, 70, 77, 80, 249] or T cells [45, 77, 159] in the included studies. IDO is a known marker of MSC immunosuppressive effects, and IDO upregulation can be correlated with inhibited T cell proliferation [187, 245]. Thus, the enzyme activity assays were often conducted in combination with proliferation assays, to reflect on the influence of metabolism on the immunomodulatory capacity of MSCs, illustrated by correlation between increased IDO activity and decreased T cell proliferation, caused by tryptophan depletion and subsequent T cell starvation and death of T cells.

Adenosine diphosphate (ADO) is a well-established immunosuppressive mediator and has been employed as a functional readout of the immunomodulatory capacity of MSCs. It is generated through the stepwise hydrolysis of extracellular adenosine triphosphate, which results in upregulated ADO in MSCs both in monoculture [252] and after coculture with NK cells [253] or Tregs [207], as measured by western blot [252] and liquid chromatography [207, 252, 253]. In response to hyperglycemic conditions, the immunogenicity of MSCs was increased, as measured by colorimetric assay [231]. These assays illustrate the ever-changing nature of MSCs and how their metabolism affects their immunomodulatory characteristics. Furthermore, changes in enzyme activity reflect the immediate function of MSCs, thus providing a different perspective that can be gained through measurement of gene and/or protein expression, as enzyme activity can be inferred directly from the functionality of MSCs.

## Endocytosis

Endocytosis assays examined the effects of MSCs on the endocytic activity of immune cells via flow cytometry [13, 14, 87, 90, 113, 133, 185, 191, 249, 254–256], microscopy [96, 97, 103, 109, 133, 213, 215, 257–259], or live-cell imaging [260]. Endocytic activity is defined as the cellular uptake of particles or other cells and was increased after interactions between MSCs and macrophages [13, 14, 96, 97, 103, 109, 113, 191, 260], dendritic cells [255], T cells [259], and PBMCs [14, 186, 249, 256, 257]. Furthermore, the number of phagocytic cells in whole blood was increased by treatment with extracellular vesicles derived from MSCs [87]. In a heterogeneous population, effective endocytic cells can be distinguished through the labelling of specific cell markers [14, 186, 249, 257]. In neutrophils, both phagocytosis and efferocytosis were increased after coculture with MSCs [90, 97, 185, 213, 215, 258]. Most of these assays investigated endocytosis by immune cells, but MSCs were able to clear apoptotic T cells through endocytosis; however, their uptake of living cells was hardly detectable [133]. Endocytosis by immune cells is often associated with their polarization towards an anti-inflammatory phenotype and can therefore be used to ensure the functionality of polarized immune cells, possibly in combination with a polarization assay.

## Cell death

Intrinsically, MSCs can regulate cell death, for example, by upregulating inflammasomes related to apoptosis, as measured by qPCR and western blot [54] but also by regulating autophagy, which was increased by treatment with proinflammatory cytokines [115] and decreased after interaction with T cells [261], as measured by transmission electron microscopy [115, 261], qPCR [261], and western blot [261]. Furthermore, both flow cytometric analysis [46, 60, 99, 127, 140, 169, 185, 213, 215, 245, 258, 261–263] and ELISA [264] revealed that MSCs prevented the apoptosis of nearby immune cells. Interaction with MSCs resulted in decreased apoptosis of PBMCs [127, 140, 169, 245, 262], T cells [60, 121, 261], macrophages [46], neutrophils [213, 215, 258], and B cells [99]. While the apoptosis of NK cells increased after coculture with MSCs, this finding was not in concordance with previous findings from similar assays [253]. Treatment of PBMCs from patients with systemic lupus erythematosus with either MSCs or MSC-derived extracellular vesicles led to increased apoptosis [237]. Similarly, increased apoptosis was observed in PBMCs from MSC‒PBMC cocultures subjected to palmitate-induced metabolic stress [264]. These findings suggest that certain pathological or stress conditions may impair the responsiveness of PBMCs to the protective effects of MSCs. While autophagy and apoptosis in MSCs are primarily indicators of cellular well-being rather than direct measures of immunological function, they can indirectly influence their immunomodulatory capacity. Assessing MSC-induced apoptosis in neighbouring immune cells, in combination with other immune assays, helps distinguish whether the observed effects arise from immune cell death or from changes in the expression of immunological markers.

## Cytotoxicity

MSCs can alter the cytotoxicity of immune cells. In NK cells [40, 118–120, 227], PBMCs [120, 220, 231], and T cells [220], cytotoxicity was decreased after coculture with MSCs, as shown by colorimetric assays [118–120], flow cytometry [40, 120, 220, 227], cell counting [231], and a Chromium-51 release assay [40]. However, the killing of target cells by PBMCs was increased after coculture with MSCs and stimulation with TLR agonists, as visualized by live-cell imaging [265].

The cytotoxic effect of neutrophils is primarily mediated through their initiation of respiratory bursts, generating reactive oxygen species crucial for the innate immune response. The effect of MSCs on respiratory bursts depended on the experimental conditions, as MSC-derived extracellular vesicles had a limited effect, whereas conditioned medium from MSCs increased respiratory burst activity, as measured by western blot [213, 215, 258]. Additionally, the combination of MSCs and alpha-1 antitrypsin inhibited respiratory bursts, as visualized by immunofluorescence microscopy [97]. These varying results may reflect the plasticity of MSCs, whereby they sense, adapt, and respond to specific phases and demands during an inflammatory response, an ability that may be vital for their prolonged survival in an inflammatory milieu.

## Migration

MSCs can regulate the recruitment of immune cells to sites of inflammation, which is important for their role as regulators of the immune response and in the activation of the immune system. Indirect coculture with MSCs upregulated both monocyte migration and the expression of genes related to monocyte recruitment, as measured by flow cytometry [11], cell counting [106], and qPCR [11]. However, exposure of MSCs to blood components resulted in reduced recruitment of monocytes, as measured by cell counting [88]. Likewise, the immunomodulatory effects of conditioned medium from MSCs were investigated by flow cytometry, which revealed that the migration of PBMCs was inhibited [58]. The expression of chemokines by MSCs via qPCR and Luminex analysis was found to be increased and inversely correlated with T cell proliferation [17]. The migration of neutrophils was increased in response to treatment with extracellular vesicles or conditioned medium from primed MSCs, as measured by live-cell imaging [87]. Examining how MSCs influence resident immune cells can provide insight into the extent of their regulatory capacity in initiating and modulating immune responses. Migration assays have frequently demonstrated enhanced recruitment of immune cells, thus contributing to a proinflammatory environment that can further amplify the immunomodulatory effects of MSCs.

## Inflammatory pathways

Small molecules such as metformin [243], chlorzoxazone [170], phytosomal curcumin [152], and suramin [93] have been used to enhance the anti-inflammatory properties of MSCs, with the aim of increasing their therapeutic efficacy in diseases such as systemic lupus erythematosus. While not strictly mechanistic studies, such interventions may provide indirect insights into key signalling pathways involved in MSC immunomodulation. Assays revealed the upregulation of anti-inflammatory pathways via western blotting [170, 243] and the downregulation of proinflammatory pathways via qPCR [152]. Proinflammatory pathways were downregulated in PBMCs derived from alopecia areata patients after coculture with MSCs [266], in macrophages cocultured with primed MSCs [93], and in macrophages from patients with coronary atherosclerotic heart disease treated with conditioned medium from autologous MSCs [46], all of which were measured via western blotting. Assays investigating inflammatory pathways can be used to discover mechanisms of action by examining the effects of specific mediators.

## Differentiation

In response to inflammation, monocytes migrate to sites of inflammation, where they differentiate into macrophages or dendritic cells depending on the surrounding tissue milieu. When monocytes were cocultured with MSCs, both their activation and ability to differentiate into either macrophages or dendritic cells were decreased, as measured by qPCR [255]. Macrophages and dendritic cells perform effector functions that are crucial for promoting the inflammatory response. However, if uncontrolled and prolonged, this may become detrimental.

## Discussion

Investigation of immunomodulatory properties of MSCs is a rapidly expanding area of research. Our literature search identified over 2000 publications since 2019 alone, underscoring the importance of evaluating the current landscape and future trajectory of this research area. MSCs are widely recognized for their immunomodulatory properties [1, 2], yet the underlying mechanisms of action remain incompletely understood. Interestingly, this review highlights that most studies still explore the characteristics of MSCs and functional assays focusing on proliferation and polarization (Fig. 2).

A critical challenge remains MSC heterogeneity, which impairs reproducibility and repeatability. Although papers that did not meet ISCT criteria [30] were excluded from this review (Fig. 1B), variability persisted and MSCs from different sources exhibited variable expression profiles and immunomodulatory potential [70, 76, 77], which has implications for consistent outcomes [2]. Furthermore, the culture conditions, such as hypoxia [57], also changed the MSC secretome and thereby their immunomodulatory effect. This complicates comparison between studies and assays and underscores the need for source-specific comparisons and transparent reporting of MSC origins. Some studies proposed standardized protocols for isolation and handling of MSCs [4, 15–17, 108, 141, 204, 205, 234, 235, 263, 267–271], thereby directly addressing the need for consensus in MSC research, which also aligns with broader initiatives within the scientific community [272]. Their efforts to create standardized and validated assays for testing MSC immunomodulatory potential based on accepted sets of guidelines from the International Conference on Harmonization and European Medicines Agency [273] and the U.S. Food and Drug Administration [274] are promising, especially regarding lymphocyte proliferation assays [4, 17, 204, 205, 234, 235, 263, 267, 271]. The guidelines suggest evaluation of assay robustness, repeatability, range, and precision, as well as specificity and selectivity of methods used for assay readout, e.g. flow cytometry. While adoption is uneven, this can help define the ability of the assay to produce reliable and reproducible results, thus ensuring comparability of MSC batches and between research groups. However, many studies still report results without disclosing critical parameters. Eliminating assay variability entirely may not be feasible, but transparent reporting of key features such as MSC source, passage, and experimental conditions is essential for reproducibility and meta-analysis. Moving forward, the field should prioritize harmonization of protocols and workflows, thus aiding development of relevant assays for evaluating therapeutic potential of MSC.

Another critical variable is the strategy used to mimic inflammatory environments. Priming with cytokines or drugs offers controlled dosing and timing, thus improving standardization, but may lack in vivo relevance due to its simplicity, and possibly does not reflect in vivo concentrations. Conversely, inclusion of immune cells increases physiological relevance but adds complexity and introduces variability. Direct co-culture enables cell–cell contact but complicates analysis while indirect methods simplify separation but exclude contact-dependent mechanisms. Emerging technologies such as single-cell sequencing may help overcome these limitations by enabling detailed analysis of individual cell contributions without physical separation. However, implementation of this technology may be hindered by the high cost. Furthermore, sequencing analyses may expose personally identifiable information, posing a risk to donor anonymity. Therefore, researchers must consider whether this violates the terms under which the tissue was obtained. Information on the priming strategy or the inclusion of immune cells is important, since so many different protocols exist, and these tend to yield different MSC responses. Future efforts to describe the mechanisms could foster the ability to selectively enhance or inhibit specific characteristics of the MSCs and may be valuable for translation to clinical applications. This may be achieved by designing more complex assays using multiple cell types, scaffolds and/or three-dimensional structures with improved physiological relevance while maintaining reproducibility. Both controlled priming of MSC, inclusion of immune cells, and more complex models can yield valuable insights, but the choice should be guided by desired outcome. If a simple model provides the required readout, added complexity is unnecessary and may hinder interpretation. Thus, complex models should be employed only when they offer clear advantages in terms of physiological relevance or understanding, as the design and analyses of these models are inherently more challenging.

Characterization assays represent the most frequently used approach in MSC immunomodulation research, likely due to their simplicity, cost-effectiveness, and ability to provide rapid information on MSC phenotype. While essential for confirming MSC identity, phenotype alone does not reliably predict immunomodulatory potency due to MSC heterogeneity and plasticity. Consequently, over-reliance on phenotypic characterization without functional validation, may hinder clinical translation, yet phenotypic markers can serve as surrogates for potency if their predictive value is sufficiently demonstrated through functional assays. As mitigation, we recommend integrated workflows that pairs phenotypic characterization with at least one functional assay. This approach provides further insights into MSC mode and mechanism of action, thus improving robustness and reliability by linking MSC identity to functional effect.

Functional assays showed greater complexity and variability in design and output measures compared to characterization assays, which complicated interpretation and reproducibility. Macrophage-related assays illustrated these challenges, and although most studies reported an anti-inflammatory shift, discrepancies persisted due to variable marker selection and differentiation protocols. Moreover, reliance on THP-1-derived macrophages raises concerns about translational validity, as cell lines may not fully reflect properties of primary cells. Additional insights could be gained through more studies using primary cells, focusing on development of reliable marker panels, and by combining characterization assays with functional assays, e.g., investigating endocytosis, assay robustness could be improved. These inconsistencies highlight the need for critical evaluation of assay performance. Notably, proliferation assays appeared to be more uniform, and commonly used buffy-coat-derived PBMCs. Despite variations in PBMC isolation and stimulation protocols, these assays consistently demonstrated MSC inhibition of PBMC and T cell proliferation and a shift towards Treg subsets with fairly consistent usage of specific markers. This reproducibility suggests that proliferation assays may serve as a benchmark for functional evaluation. Flow cytometry was extensively used, reflecting the ability to deliver high-throughput, multi-parameter, and quantitative analysis. The versatility and reproducibility of this method make it a cornerstone for functional evaluation.

Assays involving B cells, NK cells, and neutrophils were less frequent and showed greater variability, largely due to methodological differences. For B cells, technical challenges in isolation may explain their limited use. Proliferation results differed, with some reporting expansion of regulatory B cells [163] and other suppression, likely due to different activation protocols and B cell phenotype. NK cell study outcomes varied in cytotoxicity, polarization, and proliferation, which was influenced by MSC health status [40], use of the MSC secretome [40, 117], and use of cell lines instead of primary NK cells [117–120]. Neutrophil studies consistently showed increased endocytosis and reduced apoptosis but differed in released reactive oxygen species, depending on labelling of neutrophils [97] or MSC [215], and whether MSCs [213, 215], conditioned medium, or extracellular vesicles [213, 215] were used. These discrepancies highlight the impact of assay design on observed effects, underscoring the need for standardized protocols. Selecting immune cells that match the clinical indication is essential for ensuring translational relevance. More research on these less frequently used types of immune cells is needed to improve consistency of the reported results and clarify their predictive value.

Importantly, the categorization of assays in this review does not adequately reflect the synergistic value of combined approaches, and assigning papers to a single category creates a risk of oversimplification. Many studies employed multiple assay types to build a more comprehensive understanding of MSC function (Fig. 2B). For example, studies that paired flow cytometric evaluation of proliferation with investigation of IDO expression and/or activity by MSCs provided stronger evidence of immunomodulatory capacity than either approach alone [40, 117]. Finally, even when using primary human cells, in vitro systems cannot fully replicate the complexity of in vivo physiology. Thus, their ability to predict clinical efficacy should be interpreted cautiously.

## Conclusions

Understanding the potential implications of MSC immunomodulation is critical for reliable translation of in vitro findings to clinical applications. MSCs primarily act through constitutive expression of immunomodulatory markers and, when exposed to proinflammatory stimuli, adopt an anti-inflammatory phenotype, that can help alleviate inflammation. Despite significant progress, the field still faces challenges that hinder clinical translation. To address these challenges, we propose a minimum reporting checklist that includes MSC source, priming conditions, assay design, and immune cell characteristics. These attributes have been identified in this review as major causes of variation. Such a framework would improve transparency, facilitate cross-study comparisons, help identify sources of variability, and guide robust assay design. Due to the complexity of MSC interactions, an array of assays is needed to fully assess their therapeutic potential. A robust strategy would include a characterization assay to confirm the MSC phenotype, alongside a proliferation assay, which currently represents the most standardized functional assay. Additional assays should be incorporated to further elucidate the immunomodulatory properties of MSCs, e.g., targeting specific functions, along with gene and protein expression analysis to identify biomarkers relevant to preclinical and clinical settings.

## Supplementary Information

Below is the link to the electronic supplementary material.

Supplementary Material 1.

## Data Availability

The datasets used and/or analysed during the current study are available from the corresponding author on reasonable request.

## Abbreviations

ADO: Adenosine diphosphate
ELISA: Enzyme-linked immunosorbent assay
IDO: Indoleamine 2,3-dioxygenase
IFN: Interferon
IL: Interleukin
ISCT: International Society for Cellular Therapy
MLR: Mixed lymphocyte reaction
MSC: Mesenchymal stromal cell
NK: Natural Killer
PBMC: Peripheral blood mononuclear cell
PCR: Polymerase chain reaction
qPCR: Quantitative polymerase chain reaction
Th: T helper
TNF: Tumor necrosis factor
Treg: Regulatory T cells

## References

[1] Singer NG, Caplan AI. Mesenchymal stem cells: mechanisms of inflammation. Annu Rev Pathol. 2011;6(Electronic):1553–4014. 10.1146/annurev-pathol-011110-130230 21073342

[2] de Wolf C, van de Bovenkamp M, Hoefnagel M. Regulatory perspective on in vitro potency assays for human mesenchymal stromal cells used in immunotherapy. Cytotherapy. 2017;19(7):784–97. 28457740 10.1016/j.jcyt.2017.03.076

[3] Juhl M, Follin B, Christensen JP, Kastrup J, Ekblond A. Functional in vitro models of the inhibitory effect of adipose tissue-derived stromal cells on lymphocyte proliferation: improved sensitivity and quantification through flow cytometric analysis. J Immunol Methods. 2022;510:113360. 36130659 10.1016/j.jim.2022.113360

[4] Hansen SB, Hojgaard LD, Kastrup J, Ekblond A, Follin B, Juhl M. Optimizing an Immunomodulatory potency assay for mesenchymal stromal cell. Front Immunol. 2022;13:1085312. 36578497 10.3389/fimmu.2022.1085312 PMC9791065

[5] Antebi B, Asher AM, Rodriguez LA 2nd, Moore RK, Mohammadipoor A, Cancio LC. Cryopreserved mesenchymal stem cells regain functional potency following a 24-h acclimation period. J Transl Med. 2019;17(1):297. 31464641 10.1186/s12967-019-2038-5 PMC6716839

[6] Alhattab D, Jamali F, Ali D, Hammad H, Adwan S, Rahmeh R, et al. An insight into the whole transcriptome profile of four tissue-specific human mesenchymal stem cells. Regen Med. 2019;14(9):841–65. 30702025 10.2217/rme-2018-0137

[7] Lamas JR, Mucientes A, Lajas C, Fernández-Gutiérrez B, Lópiz Y, Marco F, et al. Check-control of inflammation displayed by bone marrow mesenchymal stem cells in rheumatoid arthritis patients. Immunotherapy. 2019;11(13):1107–16. 31378114 10.2217/imt-2019-0091

[8] Truong NC, Phan TNM, Huynh NT, Pham KD, Van Pham P. Interferon-gamma increases the immune modulation of umbilical cord-derived mesenchymal stem cells but decreases their chondrogenic potential. Adv Exp Med Biol. 2023;Part F1638:19–33. 10.1007/5584_2023_776 37291444

[9] Pan L, Liu C, Liu Q, Li Y, Du C, Kang X, et al. Human wharton’s jelly-derived mesenchymal stem cells alleviate Concanavalin A-induced fulminant hepatitis by repressing NF-kappaB signaling and Glycolysis. Stem Cell Res Therapy. 2021;12(1):496. 10.1186/s13287-021-02560-x PMC8427901 34503553

[10] Taha S, Volkmer E, Haas E, Alberton P, Straub T, David-Rus D, et al. Differences in the inflammatory response of white adipose tissue and adipose-derived stem cells. Int J Mol Sci. 2020;21(3):1086. 32041245 10.3390/ijms21031086 PMC7037886

[11] Lin J, Xie Z, Zhang Z, Li M, Ye G, Yu W, et al. LncRNA MRF drives the regulatory function on monocyte recruitment and polarization through HNRNPD-MCP1 axis in mesenchymal stem cells. J Biomed Sci. 2022;29(1):73. 36127734 10.1186/s12929-022-00858-3 PMC9490984

[12] Buyl K, Merimi M, Rodrigues RM, Rahmani S, Fayyad-Kazan M, Bouhtit F, et al. The immunological profile of adipose mesenchymal stromal/stem cells after cell expansion and inflammatory priming. Biomolecules. 2024;14(7):852. 39062566 10.3390/biom14070852 PMC11275169

[13] Cao C, Tarlé S, Kaigler D. Characterization of the Immunomodulatory properties of alveolar bone-derived mesenchymal stem cells. Stem Cell Res Ther. 2020;11(1):102. 32138791 10.1186/s13287-020-01605-x PMC7059346

[14] Wang J, Donohoe E, Canning A, Moosavizadeh S, Buckley F, Brennan MA, et al. Immunomodulatory function of licensed human bone marrow mesenchymal stromal cell-derived apoptotic bodies. Int Immunopharmacol. 2023;125:111096. 37871378 10.1016/j.intimp.2023.111096

[15] Christy BA, Herzig MC, Abaasah IE, Heard TC, Cap AP, Bynum JA. Refrigerated human mesenchymal stromal cells as an alternative to cryostorage for use in clinical investigation. Transfusion. 2023;63(7):1366–75. 37293980 10.1111/trf.17454

[16] Christy BA, Herzig MC, Delavan CP, Abaasah I, Cantu C, Salgado C, et al. Use of multiple potency assays to evaluate human mesenchymal stromal cells. J Trauma Acute Care Surg. 2020;89(2S Suppl 2):S109–17. 32744836 10.1097/TA.0000000000002743

[17] Lipat AJ, Cottle C, Pirlot BM, Mitchell J, Pando B, Helmly B, et al. Chemokine assay matrix defines the potency of human bone marrow mesenchymal stromal cells. Stem Cells Transl Med. 2022;11(9):971–86. 35881077 10.1093/stcltm/szac050 PMC9492268

[18] Oja S, Kaartinen T, Ahti M, Korhonen M, Laitinen A, Nystedt J. The utilization of freezing steps in mesenchymal stromal cell (MSC) manufacturing: potential impact on quality and cell functionality attributes. Front Immunol. 2019;10:1627. 31379832 10.3389/fimmu.2019.01627 PMC6646664

[19] Robb KP, Audet J, Gandhi R, Viswanathan S. Putative critical quality attribute matrix identifies mesenchymal stromal cells with potent Immunomodulatory and angiogenic fitness ranges in response to culture process parameters. Front Immunol. 2022;13:972095. 36532069 10.3389/fimmu.2022.972095 PMC9747767

[20] Kastrup J, Haack-Sørensen M, Juhl M, Harary Søndergaard R, Follin B, Drozd Lund L, et al. Cryopreserved Off-the-Shelf allogeneic Adipose-Derived stromal cells for therapy in patients with ischemic heart disease and heart Failure-A safety study. Stem Cells Transl Med. 2017;6(11):1963–71. 28880460 10.1002/sctm.17-0040 PMC6430047

[21] Mathiasen AB, Qayyum AA, Jørgensen E, Helqvist S, Fischer-Nielsen A, Kofoed KF, et al. Bone marrow-derived mesenchymal stromal cell treatment in patients with severe ischaemic heart failure: a randomized placebo-controlled trial (MSC-HF trial). Eur Heart J. 2015;36(Electronic):1522–9645. 10.1093/eurheartj/ehv136 25926562

[22] Mathiasen AB, Qayyum AA, Jørgensen E, Helqvist S, Kofoed KF, Haack-Sørensen M, et al. Bone marrow-derived mesenchymal stromal cell treatment in patients with ischaemic heart failure: final 4-year follow-up of the MSC-HF trial. Eur J Heart Fail. 2020;22(5):884–92. 31863561 10.1002/ejhf.1700

[23] Qayyum AA, Frljak S, Juhl M, Poglajen G, Zemljičl G, Cerar A, et al. Mesenchymal stromal cells to treat patients with non-ischaemic heart failure: results from SCIENCE II pilot study. ESC Heart Fail. 2024;11(6):3882–91. 39039797 10.1002/ehf2.14925 PMC11631292

[24] Qayyum AA, Lund TK, Jensen PB, Jensen K, Haack-Sørensen M, Ekblond A, et al. Allogeneic mesenchymal stromal cell therapy on primary graft dysfunction after lung transplantation. JHLT Open. 2025;8:100254. 40247997 10.1016/j.jhlto.2025.100254 PMC12005341

[25] Qayyum AA, Mathiasen AB, Helqvist S, Jørgensen E, Haack-Sørensen M, Ekblond A, et al. Autologous adipose-derived stromal cell treatment for patients with refractory angina (MyStromalCell Trial): 3-years follow-up results. J Transl Med. 2019;17(1):360. 31711513 10.1186/s12967-019-2110-1 PMC6849216

[26] Qayyum AA, Mathiasen AB, Mygind ND, Kühl JT, Jørgensen E, Helqvist S, et al. Adipose-Derived stromal cells for treatment of patients with chronic ischemic heart disease (MyStromalCell Trial): A randomized Placebo-Controlled study. Stem Cells Int. 2017;2017:5237063. 29333165 10.1155/2017/5237063 PMC5733128

[27] Qayyum AA, Mouridsen M, Nilsson B, Gustafsson I, Schou M, Nielsen OW, et al. Danish phase II trial using adipose tissue derived mesenchymal stromal cells for patients with ischaemic heart failure. ESC Heart Fail. 2023;10(2):1170–83. 36638837 10.1002/ehf2.14281 PMC10053281

[28] Qayyum AA, van Klarenbosch B, Frljak S, Cerar A, Poglajen G, Traxler-Weidenauer D, et al. Effect of allogeneic adipose tissue-derived mesenchymal stromal cell treatment in chronic ischaemic heart failure with reduced ejection fraction - the SCIENCE trial. Eur J Heart Fail. 2023;25(4):576–87. 36644821 10.1002/ejhf.2772

[29] Johnstone BH, Gu D, Lin CH, Du J, Woods EJ. Identification of a fundamental cryoinjury mechanism in MSCs and its mitigation through cell-cycle synchronization prior to freezing. Cryobiology. 2023;113:104592. 37827209 10.1016/j.cryobiol.2023.104592

[30] Dominici M, Le Blanc K, Mueller I, Slaper-Cortenbach I, Marini FC, Krause DS, et al. Minimal criteria for defining multipotent mesenchymal stromal cells. The international society for cellular therapy position statement. Cytotherapy. 2006;8(4):315–7. 16923606 10.1080/14653240600855905

[31] J. WHB. readxl: read excel files. 2025. Accessed on 05 sep 2025. https://readxl.tidyverse.org

[32] Wickham HAM, Bryan J, Chang W, McGowan LD, François R, Grolemund G, Hayes A, Henry L, Hester J, Kuhn M, Pedersen TL, Miller E, Bache SM, Müller K, Ooms J, Robinson D, Seidel DP, Spinu V, Takahashi K, Vaughan D, Wilke C, Woo K, Yutani H. Welcome to the tidyverse. J Open Source Softw. 2019;4:1686.

[33] Schauberger PWA. openxlsx: Read, Write and Edit xlsx Files. 2025.

[34] Garnier, Simon, Ross, Noam, Rudis, Robert, Camargo, Antônio Pedro, Sciaini, Marco, Scherer, Cédric. viridis(Lite) - Colorblind-Friendly Color Maps for R. 2025.

[35] Csardi G, Nepusz T.. The Igraph software package for complex network research. InterJournal. Complex Systems:1695. 2006.

[36] Bojanowski M. {intergraph}: coercion routines for network data objects. 2023.

[37] Butts CT. sna: tools for social network analysis. 2024.

[38] Schloerke B, Cook D, Larmarange J, Briatte F, Marbach M, Thoen E, Elberg A, Crowley J, GGally: extension to ‘ggplot2’2025. 2025. Accessed on 20 nov 2025. https://ggobi.github.io/ggally/

[39] Conway JR, Lex A, Gehlenborg N. UpSetR: an R package for the visualization of intersecting sets and their properties. Bioinformatics. 2017;33(18):2938–40. 28645171 10.1093/bioinformatics/btx364 PMC5870712

[40] Abomaray F, Wagner AK, Chrobok M, Ekblad-Nordberg A, Gidlof S, Alici E, et al. The effect of mesenchymal stromal cells derived from endometriotic lesions on natural killer cell function. Front Cell Dev Biol. 2021;9:612714. 34988070 10.3389/fcell.2021.612714 PMC8722454

[41] Andrzejewska A, Catar R, Schoon J, Qazi TH, Sass FA, Jacobi D, et al. Multi-Parameter analysis of biobanked human bone marrow stromal cells shows little influence for donor age and mild comorbidities on phenotypic and functional properties. Front Immunol. 2019;10:2474. 31781089 10.3389/fimmu.2019.02474 PMC6857652

[42] Cruz T, Lopez-Giraldo A, Noell G, Guirao A, Casas-Recasens S, Garcia T, et al. Smoking impairs the Immunomodulatory capacity of lung-resident mesenchymal stem cells in chronic obstructive pulmonary disease. Am J Respir Cell Mol Biol. 2019;61(5):575–83. 30978114 10.1165/rcmb.2018-0351OC

[43] Gul F, Genc D, Arslantas MK, Zibandeh N, Topcu L, Akkoc T, et al. Dental follicle mesenchymal stem cells regulate responses in sepsis. Marmara Med J. 2020;33(1):7–16.

[44] Kuca-Warnawin E, Plebanczyk M, Ciechomska M, Olesinska M, Szczesny P, Kontny E. Impact of Adipose-Derived mesenchymal stem cells (ASCs) of rheumatic disease patients on T helper cell differentiation. Int J Mol Sci. 2022;23(10):5317. 35628127 10.3390/ijms23105317 PMC9140468

[45] Kuca-Warnawin E, Kurowska W, Plebanczyk M, Wajda A, Kornatka A, Burakowski T, et al. Basic properties of Adipose-Derived mesenchymal stem cells of rheumatoid arthritis and osteoarthritis patients. Pharmaceutics. 2023;15(3):1003. 36986863 10.3390/pharmaceutics15031003 PMC10051260

[46] Li JZ, Cao TH, Han JC, Qu H, Jiang SQ, Xie BD, et al. Comparison of adipose- and bone marrow-derived stem cells in protecting against ox-LDL-induced inflammation in M1-macrophage-derived foam cells. Mol Med Rep. 2019;19(4):2660–70. 30720126 10.3892/mmr.2019.9922 PMC6423631

[47] Pang Y, Geng S, Zhang H, Lai P, Liao P, Zeng L, et al. Phenotype of mesenchymal stem cells from patients with myelodyplastic syndrome maybe partly modulated by decitabine. Oncol Lett. 2019;18(5):4457–66. 31611955 10.3892/ol.2019.10788 PMC6781515

[48] Pedrosa M, Gomes J, Laranjeira P, Duarte C, Pedreiro S, Antunes B, et al. Immunomodulatory effect of human bone marrow-derived mesenchymal stromal/stem cells on peripheral blood T cells from rheumatoid arthritis patients. J Tissue Eng Regen Med. 2020;14(1):16–28. 31502378 10.1002/term.2958

[49] Riekert M, Almanzar G, Schmalzing M, Schütze N, Jakob F, Prelog M. Mesenchymal stem cells modulate IL-17 and IL-9 production induced by Th17-inducing cytokine conditions in autoimmune arthritis: an explorative analysis. Adv Rheumatol. 2023;63(1):37. 37525265 10.1186/s42358-023-00317-z

[50] Sharma V, Rawat S, Gupta S, Tamta S, Sharma R, Seth T, et al. Human acquired aplastic anemia patients’ Bone-Marrow-Derived mesenchymal stem cells are not influenced by hematopoietic compartment and maintain stemness and immune properties. Anemia. 2021;2021:6678067. 34012684 10.1155/2021/6678067 PMC8105116

[51] Visconte C, Taiana MM, Colombini A, De Luca P, Ragni E, de Girolamo L. Donor sites and harvesting techniques affect MiRNA cargos of extracellular vesicles released by human adipose-derived mesenchymal stromal cells. Int J Mol Sci. 2024;25(12):6450. 38928156 10.3390/ijms25126450 PMC11203784

[52] Davoodi Asl F, Sahraei SS, Kalhor N, Fazaeli H, Sheykhhasan M, Soleimani Moud S, et al. Promising effects of exosomes from menstrual blood-derived mesenchymal stem cells on endometriosis. Reprod Biol. 2023;23(3):100788. 37542905 10.1016/j.repbio.2023.100788

[53] Chen X, Luo X, Wei Y, Sun H, Dai L, Tangzhou Y, et al. LncRNA H19 induces immune dysregulation of BMMSCs, at least partly, by inhibiting IL-2 production. Mol Med. 2021;27(1):61. 34130625 10.1186/s10020-021-00326-y PMC8207721

[54] Tan W, Gu Z, Leng J, Zou X, Chen H, Min F, et al. Let-7f-5p ameliorates inflammation by targeting NLRP3 in bone marrow-derived mesenchymal stem cells in patients with systemic lupus erythematosus. Biomed Pharmacother. 2019;118:109313. 31545233 10.1016/j.biopha.2019.109313

[55] Oliva-Olivera W, Castellano-Castillo D, von Meyenn F, Cardona F, Lönnberg T, Tinahones FJ. Human adipose tissue-derived stem cell paracrine networks vary according metabolic risk and after TNFα-induced death: an analysis at the single-cell level. Metabolism. 2021;116:154466. 33333081 10.1016/j.metabol.2020.154466

[56] Afsharzadeh N, Lavi Arab F, Sankian M, Samiei L, Tabasi NS, Afsharzadeh D, et al. Comparative assessment of proliferation and Immunomodulatory potential of hypericum perforatum plant and callus extracts on mesenchymal stem cells derived adipose tissue from multiple sclerosis patients. Inflammopharmacology. 2021;29(5):1399–412. 34510276 10.1007/s10787-021-00838-3

[57] Paprocka M, Kraskiewicz H, Bielawska-Pohl A, Krawczenko A, Masłowski L, Czyżewska-Buczyńska A, et al. From primary MSC culture of adipose tissue to immortalized cell line producing cytokines for potential use in regenerative medicine therapy or immunotherapy. Int J Mol Sci. 2021;22(21):11439. 34768869 10.3390/ijms222111439 PMC8584013

[58] Islam A, Urbarova I, Bruun JA, Martinez-Zubiaurre I. Large-scale secretome analyses unveil the superior immunosuppressive phenotype of umbilical cord stromal cells as compared to other adult mesenchymal stromal cells. Eur Cells Mater. 2019;37:153–74. 10.22203/eCM.v037a10 30785213

[59] Ozdemir AT, Kirmaz C, Ozdemir RBO, Oztatlici M, Sonmez PK, Tuglu MI. In-Vitro evaluation of Immunomodulation effects of mesenchymal stem Cell-Derived exosomes in refractory chronic spontaneous urticaria. Asthma Allergy Immunol. 2023;21(1):45–54.

[60] Kuca-Warnawin E, Janicka I, Szczęsny P, Olesińska M, Bonek K, Głuszko P, et al. Modulation of T-Cell activation markers expression by the adipose Tissue-Derived mesenchymal stem cells of patients with rheumatic diseases. Cell Transpl. 2020;29:963689720945682. 10.1177/0963689720945682 PMC7784571 32878464

[61] Baharlou R, Rashidi N, Ahmadi-Vasmehjani A, Khoubyari M, Sheikh M, Erfanian S. Immunomodulatory effects of human adipose tissue-derived mesenchymal stem cells on T cell subsets in patients with rheumatoid arthritis. Iran J Allergy Asthma Immunol. 2019;18(1):114–9. 30848580

[62] Breitman M, Bonfield TL, Caplan AI, Lazarus HM, Haghiac M, LaSalvia S, et al. Optimization of human mesenchymal stem cells for rheumatoid arthritis: implications for improved therapeutic outcomes. ACR Open Rheumatol. 2022;4(2):152–60. 34792869 10.1002/acr2.11356 PMC8843759

[63] Kuca-Warnawin E, Skalska U, Janicka I, Musialowicz U, Bonek K, Gluszko P, et al. The phenotype and secretory activity of adipose-derived mesenchymal stem cells (ASCs) of patients with rheumatic diseases. Cells. 2019;8(12):1659. 31861245 10.3390/cells8121659 PMC6952982

[64] Kuca-Warnawin E, Janicka I, Bonek K, Kontny E. Modulatory impact of adipose-derived mesenchymal stem cells of ankylosing spondylitis patients on t helper cell differentiation. Cells. 2021;10(2):1–15. 10.3390/cells10020280 PMC7912699 33573252

[65] Loisel S, Lansiaux P, Rossille D, Ménard C, Dulong J, Monvoisin C, et al. Regulatory B cells contribute to the clinical response after bone Marrow-Derived mesenchymal stromal cell infusion in patients with systemic sclerosis. Stem Cells Transl Med. 2023;12(4):194–206. 36928395 10.1093/stcltm/szad010 PMC10108721

[66] McCoy SS, Giri J, Das R, Paul PK, Pennati A, Parker M, et al. Minor salivary gland mesenchymal stromal cells derived from patients with Sjӧgren’s syndrome deploy intact immune plasticity. Cytotherapy. 2021;23(4):301–10. 10.1016/j.jcyt.2020.09.008 PMC8728747 33262072

[67] Helsper S, Yuan X, Bagdasarian FA, Athey J, Li Y, Borlongan CV, et al. Multinuclear MRI reveals early efficacy of stem cell therapy in stroke. Transl Stroke Res. 2023;14(4):545–61. 35900719 10.1007/s12975-022-01057-w PMC10733402

[68] Aliakbari S, Mohammadi M, Rezaee MA, Amini AA, Fakhari S, Rahmani MR. Impaired immunomodulatory ability of type 2 diabetic adipose-derived mesenchymal stem cells in regulation of inflammatory condition in mixed leukocyte reaction. EXCLI J. 2019;18:852–65. 31645845 10.17179/excli2019-1575 PMC6806137

[69] Hickson LJ, Eirin A, Conley SM, Taner T, Bian X, Saad A, et al. Diabetic kidney disease alters the transcriptome and function of human Adipose-Derived mesenchymal stromal cells but maintains Immunomodulatory and paracrine activities important for renal repair. Diabetes. 2021;70(7):1561–74. 33858824 10.2337/db19-1268 PMC8336004

[70] Sineh Sepehr K, Razavi A, Hassan ZM, Fazel A, Abdollahpour-Alitappeh M, Mossahebi-Mohammadi M, et al. Comparative Immunomodulatory properties of mesenchymal stem cells derived from human breast tumor and normal breast adipose tissue. Cancer Immunol Immunother. 2020;69(9):1841–54. 32350594 10.1007/s00262-020-02567-y PMC11027656

[71] Krueger TE, Thorek DLJ, Meeker AK, Isaacs JT, Brennen WN. Tumor-infiltrating mesenchymal stem cells: drivers of the immunosuppressive tumor microenvironment in prostate cancer? Prostate. 2019;79(3):320–30. 30488530 10.1002/pros.23738 PMC6549513

[72] Schweizer R, Waldner M, Oksuz S, Zhang W, Komatsu C, Plock JA, et al. Evaluation of Porcine versus human mesenchymal stromal cells from three distinct donor locations for cytotherapy. Front Immunol. 2020;11:826. 32435248 10.3389/fimmu.2020.00826 PMC7218165

[73] Haghighitalab A, Matin MM, Amin A, Minaee S, Bidkhori HR, Doeppner TR, et al. Investigating the effects of IDO1, PTGS2, and TGF-β1 overexpression on Immunomodulatory properties of hTERT-MSCs and their extracellular vesicles. Sci Rep. 2021;11(1):7825. 33837229 10.1038/s41598-021-87153-7 PMC8035148

[74] Abu-El-Rub E, Khaswaneh RR, Almahasneh FA, Almazari R, Alzu’bi A. Adipose tissue and bone marrow-derived mesenchymal stem cells are not really the same: investigating the differences in their immunomodulatory, migratory, and adhesive profile. Biochem Genet. 2024;63:378–92. 38441812 10.1007/s10528-024-10724-6

[75] Burja B, Barlic A, Erman A, Mrak-Poljsak K, Tomsic M, Sodin-Semrl S, et al. Human mesenchymal stromal cells from different tissues exhibit unique responses to different inflammatory stimuli. Curr Res Translational Med. 2020;68(4):217–24. 10.1016/j.retram.2020.05.006 32843323

[76] Jafari M, Asghari A, Delbandi AA, Jalessi M, Jazayeri MH, Samarei R, et al. Priming TLR3 and TLR4 in human adipose- and olfactory mucosa-derived mesenchymal stromal cells and comparison of their cytokine secretions. Cytotechnology. 2020;72(1):57–68. 31898754 10.1007/s10616-019-00357-8 PMC7002628

[77] Torres Crigna A, Uhlig S, Elvers-Hornung S, Klüter H, Bieback K. Human adipose tissue-derived stromal cells suppress human, but not murine lymphocyte proliferation, via indoleamine 2,3-dioxygenase activity. Cells. 2020;9(11):2419. 33167329 10.3390/cells9112419 PMC7694333

[78] Kang JY, Oh MK, Joo H, Park HS, Chae DH, Kim J, et al. Xeno-free condition enhances therapeutic functions of human wharton’s jelly-derived mesenchymal stem cells against experimental colitis by upregulated indoleamine 2,3-dioxygenase activity. J Clin Med. 2020;9(9):1–20. 10.3390/jcm9092913 PMC7565923 32927587

[79] Özgül Özdemir RB, Özdemir AT, Kırmaz C, Eker Sarıboyacı A, Karaöz E, Erman G, et al. Age-related changes in the Immunomodulatory effects of human dental pulp derived mesenchymal stem cells on the CD4(+) T cell subsets. Cytokine. 2021;138:155367. 33223447 10.1016/j.cyto.2020.155367

[80] Herzig MC, Christy BA, Montgomery RK, Delavan CP, Jensen KJ, Lovelace SE, et al. Interactions of human mesenchymal stromal cells with peripheral blood mononuclear cells in a mitogenic proliferation assay. J Immunol Methods. 2021;492:113000. 33609532 10.1016/j.jim.2021.113000

[81] Meenakshi Sundaram R, Kadapakkam Nandabalan S, Rupert S, Srinivasan P, Sankar P, Patra B, et al. Differential Immunomodulation of human mesenchymal stromal cells from various sources in an inflammation mimetic milieu. Cytotherapy. 2022;24(2):110–23. 34740526 10.1016/j.jcyt.2021.09.005

[82] Porter AP, Pirlot BM, Dyer K, Uwazie CC, Nguyen J, Turner C, et al. Conglomeration of T- and B-Cell matrix responses determines the potency of human bone marrow mesenchymal stromal cells. Stem Cells. 2022;40(12):1134–48. 36056823 10.1093/stmcls/sxac064

[83] Suzdaltseva Y, Goryunov K, Silina E, Manturova N, Stupin V, Kiselev SL. Equilibrium among inflammatory factors determines human MSC-Mediated immunosuppressive effect. Cells. 2022;11(7):1210. 35406773 10.3390/cells11071210 PMC8997511

[84] Bi Y, Lin X, Liang H, Yang D, Zhang X, Ke J, et al. Human adipose Tissue-Derived mesenchymal stem cells in parkinson’s disease: Inhibition of T helper 17 cell differentiation and regulation of immune balance towards a regulatory T cell phenotype. Clin Interv Aging. 2020;15:1383–91. 32884248 10.2147/CIA.S259762 PMC7434526

[85] Lee HJ, Jung H, Kim DK. IDO and CD40 May be key molecules for Immunomodulatory capacity of the primed tonsil-derived mesenchymal stem cells. Int J Mol Sci. 2021;22(11):5772. 34071285 10.3390/ijms22115772 PMC8198434

[86] Lotfinejad P, Shamsasenjan K, Baradaran B, Safarzadeh E, Kazemi T, Movassaghpour AA. Immunomodulatory effect of human umbilical cord Blood-derived mesenchymal stem cells on activated T-lymphocyte. Iran J Allergy Asthma Immunol. 2021;20(6):711–20. 34920654 10.18502/ijaai.v20i6.8022

[87] Calligaris M, Zito G, Busa R, Bulati M, Iannolo G, Gallo A, et al. Proteomic analysis and functional validation reveal distinct therapeutic capabilities related to priming of mesenchymal stromal/stem cells with IFN-gamma and hypoxia: potential implications for their clinical use. Front Cell Dev Biol. 2024;12:1385712. 38882056 10.3389/fcell.2024.1385712 PMC11179434

[88] Davies LC, Queckbörner S, Jylhä CE, Andrén AT, Forshell TZP, Blanc KL. Lysis and phenotypic modulation of mesenchymal stromal cells upon blood contact triggers anti-inflammatory skewing of the peripheral innate immune repertoire. Cytotherapy. 2023;25(9):956–66. 37354149 10.1016/j.jcyt.2023.05.009

[89] Al-Azab M, Walana W, Wei J, Li W, Tang Y, Wei X, et al. TL1A/TNFR2 axis enhances immunoregulatory effects of bone marrow derived mesenchymal stem cell by Indian Hedgehog signaling pathway. Int J Stem Cells. 2021;14(1):58–73. 33122466 10.15283/ijsc19121 PMC7904531

[90] Amaro-Prellezo E, Gomez-Ferrer M, Hakobyan L, Ontoria-Oviedo I, Peiro-Molina E, Tarazona S, et al. Extracellular vesicles from dental pulp mesenchymal stem cells modulate macrophage phenotype during acute and chronic cardiac inflammation in athymic nude rats with myocardial infarction. Inflamm Regeneration. 2024;44(1):25. 10.1186/s41232-024-00340-7 PMC11134765 38807194

[91] Caldwell AS, Rao VV, Golden AC, Bell DJ, Grim JC, Anseth KS. Mesenchymal stem cell-inspired microgel scaffolds to control macrophage polarization. Bioeng Translational Med. 2021;6(2):e10217. 10.1002/btm2.10217 PMC8126823 34027099

[92] Liebmann K, Castillo MA, Jergova S, Best TM, Sagen J, Kouroupis D. Modification of mesenchymal Stem/Stromal Cell-Derived small extracellular vesicles by calcitonin gene related peptide (CGRP) antagonist: potential implications for inflammation and pain reversal. Cells. 2024;13(6):484. 38534328 10.3390/cells13060484 PMC10969778

[93] Arabiyat AS, Yeisley DJ, Güiza-Argüello VR, Qureshi F, Culibrk RA, Hahn J, et al. Effects of stromal cell conditioned medium and antipurinergic treatment on macrophage phenotype. Tissue Eng Part C Methods. 2022;28(12):656–71. 36329666 10.1089/ten.tec.2022.0123 PMC9807257

[94] Chen L, Merkhan MM, Forsyth NR, Wu P. Chorionic and amniotic membrane-derived stem cells have distinct, and gestational diabetes mellitus independent, proliferative, differentiation, and Immunomodulatory capacities. Stem Cell Res. 2019;40:101537. 31422237 10.1016/j.scr.2019.101537

[95] Dedier M, Magne B, Nivet M, Banzet S, Trouillas M. Anti-inflammatory effect of interleukin-6 highly enriched in secretome of two clinically relevant sources of mesenchymal stromal cells. Front Cell Dev Biol. 2023;11:1244120. 37745306 10.3389/fcell.2023.1244120 PMC10512713

[96] Gonzalez H, McCarthy S, Masterson C, Byrnes D, Sallent I, Horan E, et al. Nebulised mesenchymal stem cell derived extracellular vesicles ameliorate E. coli induced pneumonia in a rodent model. Stem Cell Res Therapy. 2023;14(1):151. 10.1186/s13287-023-03385-6 PMC10245544 37280647

[97] Han L, Wu X, Wang O, Luan X, Velander WH, Aynardi M, et al. Mesenchymal stromal cells and alpha-1 antitrypsin have a strong synergy in modulating inflammation and its resolution. Theranostics. 2023;13(9):2843–62. 37284443 10.7150/thno.83942 PMC10240832

[98] He F, Umrath F, Reinert S, Alexander D. Jaw periosteum-derived mesenchymal stem cells regulate thp-1-derived macrophage polarization. Int J Mol Sci. 2021;22(9):4310. 33919221 10.3390/ijms22094310 PMC8122347

[99] He Y, Yang S, Liu P, Li K, Jin K, Becker R, et al. Acoustofluidic interfaces for the Mechanobiological secretome of MSCs. Nat Commun. 2023;14(1):7639. 37993431 10.1038/s41467-023-43239-6 PMC10665559

[100] Jeske R, Yuan X, Fu Q, Bunnell BA, Logan TM, Li Y. In vitro culture expansion shifts the immune phenotype of human Adipose-Derived mesenchymal stem cells. Front Immunol. 2021;12:621744. 33777002 10.3389/fimmu.2021.621744 PMC7988085

[101] Li FC, Hussein H, Magalhaes M, Selvaganapathy PR, Kishen A. Deciphering stem cell from apical Papilla-Macrophage choreography using a novel 3-dimensional organoid system. J Endod. 2022;48(8):1063–e727. 35513088 10.1016/j.joen.2022.04.011

[102] Li H, Wang W, Chang J. Calcium silicate enhances immunosuppressive function of MSCs to indirectly modulate the polarization of macrophages. Regenerative Biomaterials. 2021;8(6):rbab056. 34804588 10.1093/rb/rbab056 PMC8597971

[103] Li N, Gao Z, Zhao L, Du B, Ma B, Nian H, et al. MSC-Derived small extracellular vesicles attenuate autoimmune dacryoadenitis by promoting M2 macrophage polarization and inducing Tregs via miR-100-5p. Front Immunol. 2022;13:888949. 35874782 10.3389/fimmu.2022.888949 PMC9298967

[104] Liu J, Wang H, Zhang L, Li X, Ding X, Ding G, et al. Periodontal ligament stem cells promote polarization of M2 macrophages. J Leukoc Biol. 2022;111(6):1185–97. 34982483 10.1002/JLB.1MA1220-853RR

[105] Massaro F, Corrillon F, Stamatopoulos B, Dubois N, Ruer A, Meuleman N, et al. Age-related changes in human bone marrow mesenchymal stromal cells: morphology, gene expression profile, Immunomodulatory activity and MiRNA expression. Front Immunol. 2023;14:1267550. 38130717 10.3389/fimmu.2023.1267550 PMC10733451

[106] Ogle ME, Doron G, Levy MJ, Temenoff JS. Hydrogel culture surface stiffness modulates mesenchymal stromal cell secretome and alters senescence. Tissue Eng Part A. 2020;26(23–24):1259–71. 32628570 10.1089/ten.tea.2020.0030

[107] Ren J, Szombath G, Vitale-Cross L, Stroncek DF, Robey PG, Hajdara A, et al. The potential use of THP-1, a monocytic leukemia cell Line, to predict immune-suppressive potency of human bone-marrow stromal cells (BMSCs) in vitro: a pilot study. Int J Mol Sci. 2023;24(17):13258. 37686058 10.3390/ijms241713258 PMC10488111

[108] Sadeghi S, Nimtz L, Niebergall-Roth E, Norrick A, Hägele S, Vollmer L, et al. Potency assay to predict the anti-inflammatory capacity of a cell therapy product for macrophage-driven diseases: overcoming the challenges of assay development and validation. Cytotherapy. 2024;26(5):512–23. 38441512 10.1016/j.jcyt.2024.02.004 PMC11065629

[109] Song A, Wang J, Tong Y, Fang J, Zhang Y, Zhang H, et al. BKCa channels regulate the Immunomodulatory properties of WJ-MSCs by affecting the exosome protein profiles during the inflammatory response. Stem Cell Res Ther. 2020;11(1):440. 33059770 10.1186/s13287-020-01952-9 PMC7560248

[110] Sun W, Yan S, Yang C, Yang J, Wang H, Li C, et al. Mesenchymal stem cells-derived exosomes ameliorate lupus by inducing M2 macrophage polarization and regulatory T cell expansion in MRL/lpr mice. Immunol Invest. 2022;51(6):1785–803. 35332841 10.1080/08820139.2022.2055478

[111] Teng SW, Sung HY, Wen YC, Chen SY, Lovel R, Chang WY, et al. Potential surrogate quantitative Immunomodulatory potency assay for monitoring human umbilical cord-derived mesenchymal stem cells production. Cell Biol Int. 2021;45(5):1072–81. 33470478 10.1002/cbin.11553

[112] Wei ST, Huang YC, Chiang JY, Lin CC, Lin YJ, Shyu WC, et al. Gain of CXCR7 function with mesenchymal stem cell therapy ameliorates experimental arthritis via enhancing tissue regeneration and Immunomodulation. Stem Cell Res Therapy. 2021;12(1):314. 10.1186/s13287-021-02402-w PMC8164772 34051857

[113] Üçal M, Maurer C, Etschmaier V, Hamberger D, Grünbacher G, Tögl L, et al. Inflammatory Pre-Conditioning of Adipose-Derived stem cells with cerebrospinal fluid from traumatic brain injury patients alters the Immunomodulatory potential of ADSC secretomes. J Neurotrauma. 2021;38(16):2311–22. 33514282 10.1089/neu.2020.7017

[114] Yuan X, Sun L, Jeske R, Nkosi D, York SB, Liu Y, et al. Engineering extracellular vesicles by three-dimensional dynamic culture of human mesenchymal stem cells. J Extracell Vesicles. 2022;11(6):e12235. 35716062 10.1002/jev2.12235 PMC9206229

[115] Zhou W, Li L, Tao J, Ma C, Xie Y, Ding L, et al. Autophagy Inhibition restores CD200 expression under IL-1beta microenvironment in placental mesenchymal stem cells of fetal origin and improves its pulmonary fibrosis therapeutic potential. Mol Immunol. 2022;151:29–40. 36075140 10.1016/j.molimm.2022.08.014

[116] Zhu D, Chen F, Qiang H, Qi H. SPA inhibits hBMSC osteogenic differentiation and M1 macrophage polarization by suppressing SETD2 in acute suppurative osteomyelitis. Sci Rep. 2024;14(1):12728. 38830934 10.1038/s41598-024-63219-0 PMC11148074

[117] Ko E, Yoon T, Lee Y, Kim J, Park YB. ADSC secretome constrains NK cell activity by attenuating IL-2-mediated JAK-STAT and AKT signaling pathway via upregulation of CIS and DUSP4. Stem Cell Res Ther. 2023;14(1):329. 37964351 10.1186/s13287-023-03516-z PMC10648656

[118] Ozdemir AT, Kirmaz C, Ozdemir RBO, Degirmenci P, Oztatlici M, Degirmenci M. In-vitro evaluation of effects of mesenchymal stem cells on tlr3, tlr7/8 and tlr9-activated natural killer cells. Eurasian J Med Oncol. 2021;5(1):71–9.

[119] Wu R, Soland M, Liu G, Shi Y, Zhang C, Tang Y, et al. Functional characterization of the Immunomodulatory properties of human urine-derived stem cells. Translational Androl Urol. 2021;10(9):3566–78. 10.21037/tau-21-506 PMC8511544 34733653

[120] Yi E, Go J, Yun SH, Lee SE, Kwak J, Kim SW, et al. CEACAM1-engineered MSCs have a broad spectrum of Immunomodulatory functions and therapeutic potential via cell-to-cell interaction. Biomaterials. 2024;311:122667. 38878480 10.1016/j.biomaterials.2024.122667

[121] Li M, Soder R, Abhyankar S, Abdelhakim H, Braun MW, Trinidad CV, et al. WJMSC-derived small extracellular vesicle enhance T cell suppression through PD-L1. J Extracell Vesicles. 2021;10(4):e12067. 33598108 10.1002/jev2.12067 PMC7869022

[122] Malvicini R, De Lazzari G, Tolomeo AM, Santa-Cruz D, Ullah M, Cirillo C, et al. Influence of the isolation method on characteristics and functional activity of mesenchymal stromal cell-derived extracellular vesicles. Cytotherapy. 2024;26(2):157–70. 38069981 10.1016/j.jcyt.2023.11.001

[123] Abu-Shahba N, Mahmoud M, Abdel-Rasheed M, Darwish Y, AbdelKhaliq A, Mohammed E, et al. Immunomodulatory and antioxidative potentials of adipose-derived mesenchymal stem cells isolated from breast versus abdominal tissue: a comparative study. Cell Regeneration. 2020;9(1):18. 33020894 10.1186/s13619-020-00056-2 PMC7536259

[124] Ahmed SM, Elkhenany HA, Ahmed TA, Ghoneim NI, Elkodous MA, Mohamed RH, et al. Diabetic microenvironment deteriorates the regenerative capacities of adipose mesenchymal stromal cells. Diabetol Metabolic Syndrome. 2024;16(1):131. 10.1186/s13098-024-01365-1 PMC11181634 38880916

[125] Barpour N, Ghorbani M, Baradaran B, Jodari-Mohammadpour Z, Nejati-Koshki K, Abdollahpour-Alitappeh M, et al. Development of an injectable chitosan-based hydrogel containing nano-hydroxy-apatite and alendronate for MSC-based therapy. Int J Biol Macromol. 2024;261:129737. 38286373 10.1016/j.ijbiomac.2024.129737

[126] Gong X, Sun D, Li Z, Shi Q, Li D, Ju X. Three-Dimensional culture of umbilical cord mesenchymal stem cells effectively promotes platelet recovery in immune thrombocytopenia. Biol Pharm Bull. 2020;43(7):1052–60. 32321879 10.1248/bpb.b19-01069

[127] Kang H, Feng J, Peng Y, Liu Y, Yang Y, Wu Y, et al. Human mesenchymal stem cells derived from adipose tissue showed a more robust effect than those from the umbilical cord in promoting corneal graft survival by suppressing lymphangiogenesis. Stem Cell Res Ther. 2023;14(1):328. 37957770 10.1186/s13287-023-03559-2 PMC10644560

[128] Kouroupis D, Bowles AC, Best TM, Kaplan LD, Correa D. CD10/Neprilysin enrichment in infrapatellar fat pad-Derived mesenchymal stem cells under Regulatory-Compliant conditions: implications for efficient synovitis and fat pad fibrosis reversal. Am J Sports Med. 2020;48(8):2013–27. 32427493 10.1177/0363546520917699

[129] Lee S, Kim HS, Min BH, Kim BG, Kim SA, Nam H, et al. Enhancement of anti-inflammatory and Immunomodulatory effects of adipose-derived human mesenchymal stem cells by making uniform spheroid on the new nano-patterned plates. Biochem Biophys Res Commun. 2021;552:164–9. 33751933 10.1016/j.bbrc.2021.03.026

[130] Madel RJ, Börger V, Dittrich R, Bremer M, Tertel T, Phuong NNT, et al. Independent human mesenchymal stromal cell-derived extracellular vesicle preparations differentially attenuate symptoms in an advanced murine graft-versus-host disease model. Cytotherapy. 2023;25(8):821–36. 37055321 10.1016/j.jcyt.2023.03.008

[131] Palma MB, Luzzani C, Andrini LB, Riccillo F, Buero G, Pelinski P, et al. Wound healing by allogeneic transplantation of specific subpopulation from human umbilical cord mesenchymal stem cells. Cell Transpl. 2021;30:963689721993774. 10.1177/0963689721993774 PMC8120529 33975446

[132] Silva-Carvalho A, Neves FAR, Saldanha-Araujo F. The immunosuppressive mechanisms of mesenchymal stem cells are differentially regulated by platelet poor plasma and fetal bovine serum supplemented media. Int Immunopharmacol. 2020;79:106172. 31926480 10.1016/j.intimp.2019.106172

[133] Zhang Z, Huang S, Wu S, Qi J, Li W, Liu S, et al. Clearance of apoptotic cells by mesenchymal stem cells contributes to immunosuppression via PGE2. EBioMedicine. 2019;45:341–50. 31248835 10.1016/j.ebiom.2019.06.016 PMC6642220

[134] Bai X, Chen T, Li Y, Ge X, Qiu C, Gou H, et al. PD-L1 expression levels in mesenchymal stromal cells predict their therapeutic values for autoimmune hepatitis. Stem Cell Res Ther. 2023;14(1):370. 38111045 10.1186/s13287-023-03594-z PMC10729378

[135] Chen H, Wen X, Liu S, Sun T, Song H, Wang F, et al. Dissecting heterogeneity reveals a unique BAMBIhigh MFGE8high subpopulation of human UC-MSCs. Adv Sci. 2022;10:e2202510. 10.1002/advs.202202510 PMC9811468 36373720

[136] Ganguly A, Swaminathan G, Garcia-Marques F, Regmi S, Yarani R, Primavera R, et al. Integrated transcriptome-proteome analyses of human stem cells reveal source-dependent differences in their regenerative signature. Stem Cell Rep. 2023;18(1):190–204. 10.1016/j.stemcr.2022.11.006 PMC9860079 36493779

[137] Li J, Wang Q, An Y, Chen X, Xing Y, Deng Q, et al. Integrative Single-Cell RNA-Seq and ATAC-Seq analysis of mesenchymal Stem/Stromal cells derived from human placenta. Front Cell Dev Biology. 2022;10:836887. 10.3389/fcell.2022.836887 PMC9017713 35450295

[138] Miceli V, Pampalone M, Vella S, Carreca AP, Amico G, Conaldi PG. Comparison of immunosuppressive and angiogenic properties of human amnion-derived mesenchymal stem cells between 2D and 3D culture systems. Stem Cells Int. 2019;2019:7486279. 30911299 10.1155/2019/7486279 PMC6397962

[139] Neo SH, Her Z, Othman R, Tee CA, Ong LC, Wang Y, et al. Expansion of human bone marrow-derived mesenchymal stromal cells with enhanced Immunomodulatory properties. Stem Cell Res Ther. 2023;14(1):259. 37726837 10.1186/s13287-023-03481-7 PMC10510228

[140] Taner T, Abrol N, Park WD, Hansen MJ, Gustafson MP, Lerman LO, et al. Phenotypic, Transcriptional, and functional analysis of liver mesenchymal stromal cells and their Immunomodulatory properties. Liver Transpl. 2020;26(4):549–63. 31950576 10.1002/lt.25718

[141] Shaz BH, Schäfer R, Fontaine MJ, Norris PJ, McKenna DH, Jin P, et al. Local manufacturing processes contribute to variability in human mesenchymal stromal cell expansion while growth media supplements contribute to variability in gene expression and cell function: a biomedical excellence for safer transfusion (BEST) Collabo. Cytotherapy. 2024;26(6):531–9. 38043052 10.1016/j.jcyt.2023.11.003

[142] Davies R, Allen S, Mennan C, Platt M, Wright K, Kehoe O. Extracellular vesicle depletion protocols of foetal bovine serum influence umbilical cord mesenchymal stromal cell Phenotype, Immunomodulation, and particle release. Int J Mol Sci. 2023;24(11):9242. 37298194 10.3390/ijms24119242 PMC10252846

[143] Erol Bozkurt A, Sel FA, Suleymanoglu M, Demirayak G, Kuruca DS, Oguz FS. The cytokine levels of cord blood- and Wharton’s Jelly-derived mesenchymal stem cells from early to late passages. Cell Biochem Biophys. 2024;82(4):3345–50. 39018006 10.1007/s12013-024-01416-4

[144] Kiselevskii MV, Vlasenko RY, Stepanyan NG, Shubina IZ, Sitdikova SM, Kirgizov KI, et al. Secretome of mesenchymal bone marrow stem cells: is it immunosuppressive or proinflammatory? Bull Exp Biol Med. 2021;172(2):250–3. 34855084 10.1007/s10517-021-05371-5 PMC8636784

[145] Nakao M, Nagase K. Harvesting methods of umbilical cord-derived mesenchymal stem cells from culture modulate cell properties and functions. Regen Ther. 2024;26:80–8. 38841206 10.1016/j.reth.2024.05.010 PMC11152751

[146] Bauer FN, Tertel T, Stambouli O, Wang C, Dittrich R, Staubach S, et al. CD73 activity of mesenchymal stromal cell-derived extracellular vesicle preparations is detergent-resistant and does not correlate with Immunomodulatory capabilities. Cytotherapy. 2023;25(2):138–47. 36244910 10.1016/j.jcyt.2022.09.006

[147] Gao Y, Chi Y, Chen Y, Wang W, Li H, Zheng W, et al. Multi-omics analysis of human mesenchymal stem cells shows cell aging that alters Immunomodulatory activity through the downregulation of PD-L1. Nat Commun. 2023;14(1):4373. 37474525 10.1038/s41467-023-39958-5 PMC10359415

[148] Al-Sharabi N, Gruber R, Sanz M, Mohamed-Ahmed S, Kristoffersen EK, Mustafa K, et al. Proteomic analysis of mesenchymal stromal cells secretome in comparison to Leukocyte- and Platelet-Rich fibrin. Int J Mol Sci. 2023;24(17):13057. 37685865 10.3390/ijms241713057 PMC10487446

[149] Canas-Arboleda M, Beltran K, Medina C, Camacho B, Salguero G. Human platelet lysate supports efficient expansion and stability of wharton’s jelly mesenchymal stromal cells via active uptake and release of soluble regenerative factors. Int J Mol Sci. 2020;21(17):1–19. 10.3390/ijms21176284 PMC7503902 32877987

[150] Dietrich I, Girdlestone J, Giele H. Differential cytokine expression in direct and indirect co-culture of Islets and mesenchymal stromal cells. Cytokine. 2022;150:155779. 34923221 10.1016/j.cyto.2021.155779

[151] Peshkova M, Korneev A, Suleimanov S, Vlasova II, Svistunov A, Kosheleva N, et al. MSCs’ conditioned media cytokine and growth factor profiles and their impact on macrophage polarization. Stem Cell Res Ther. 2023;14(1):142. 37231519 10.1186/s13287-023-03381-w PMC10214600

[152] Ayadilord M, Nasseri S, Emadian Razavi F, Saharkhiz M, Rostami Z, Naseri M. Immunomodulatory effects of phytosomal Curcumin on related-micro RNAs, CD200 expression and inflammatory pathways in dental pulp stem cells. Cell Biochem Funct. 2021;39(7):886–95. 34235754 10.1002/cbf.3659

[153] Hezam K, Fu E, Zhang J, Li Z. Therapeutic trends of priming mesenchymal stem cells: A bibliometric analysis. Biochem Biophys Rep. 2024;38:2405–5808. 10.1016/j.bbrep.2024.101708 PMC11016583 38623536

[154] Saldana L, Bensiamar F, Valles G, Mancebo FJ, Garcia-Rey E, Vilaboa N. Immunoregulatory potential of mesenchymal stem cells following activation by macrophage-derived soluble factors. Stem Cell Res Therapy. 2019;10(1):58. 10.1186/s13287-019-1156-6 PMC6375172 30760316

[155] Cai S, Fan C, Xie L, Zhong H, Li A, Lv S, et al. Single-cell RNA sequencing reveals the potential mechanism of heterogeneity of Immunomodulatory properties of foreskin and umbilical cord mesenchymal stromal cells. Cell Bioscience. 2022;12(1):115. 35869528 10.1186/s13578-022-00848-w PMC9306236

[156] Du-Rocher B, Binato R, de-Freitas-Junior JCM, Corrêa S, Mencalha AL, Morgado-Díaz JA, et al. IL-17 triggers invasive and migratory properties in human MSCs, while IFNy favors their immunosuppressive capabilities: implications for the licensing process. Stem Cell Rev Rep. 2020;16(6):1266–79. 33067729 10.1007/s12015-020-10051-4 PMC7667142

[157] He H, Takahashi A, Mukai T, Hori A, Narita M, Tojo A, et al. The Immunomodulatory effect of triptolide on mesenchymal stromal cells. Front Immunol. 2021;12:686356. 34484183 10.3389/fimmu.2021.686356 PMC8415460

[158] Sakha YA, Ehsani E, Roshandel E, Jalili A, Vahdani N, Hajifathali A. Assessment of the effect of Infliximab on Immunomodulation properties of mesenchymal stem cells in vitro. Adv Pharm Bull. 2021;11(4):739–45. 34888221 10.34172/apb.2021.083 PMC8642797

[159] Behm C, Blufstein A, Gahn J, Nemec M, Moritz A, Rausch-Fan X, et al. Cytokines differently define the Immunomodulation of mesenchymal stem cells from the periodontal ligament. Cells. 2020;9(5):1222. 32423044 10.3390/cells9051222 PMC7290931

[160] Behm C, Miłek O, Rausch-Fan X, Moritz A, Andrukhov O. Paracrine- and cell-contact-mediated Immunomodulatory effects of human periodontal ligament-derived mesenchymal stromal cells on CD4(+) T lymphocytes. Stem Cell Res Ther. 2024;15(1):154. 38816862 10.1186/s13287-024-03759-4 PMC11141051

[161] Zhang Y, Lv P, Li Y, Cheng C, Hao H, Yue H. Comparison of the biological characteristics of umbilical cord mesenchymal stem cells derived from the human heterosexual twins. Differentiation. 2020;114:1–12. 32460139 10.1016/j.diff.2020.05.005

[162] Aleahmad M, Bozorgmehr M, Nikoo S, Ghanavatinejad A, Shokri MR, Montazeri S, et al. Endometrial mesenchymal stem/stromal cells: the enigma to code messages for generation of functionally active regulatory T cells. Stem Cell Res Ther. 2021;12(1):536. 34627370 10.1186/s13287-021-02603-3 PMC8502414

[163] Chen X, Cai C, Xu D, Liu Q, Zheng S, Liu L, et al. Human mesenchymal stem cell-treated regulatory CD23 + CD43+ B cells alleviate intestinal inflammation. Theranostics. 2019;9(16):4633–47. 31367246 10.7150/thno.32260 PMC6643430

[164] Song Y, Lim JY, Lim T, Im KI, Kim N, Nam YS, et al. Human mesenchymal stem cells derived from umbilical cord and bone marrow exert Immunomodulatory effects in different mechanisms. World J Stem Cells. 2020;12(9):1032–49. 33033563 10.4252/wjsc.v12.i9.1032 PMC7524695

[165] Cho WJ, Pulimamidi VK, Mittal SK, Chauhan SK. Mesenchymal stromal cells protect tissues from Th1 immune responses via IL-11 secretion. FASEB J. 2024;38(10):e23683. 38758184 10.1096/fj.202400078R PMC11149610

[166] Salimiyan S, Mohammadi M, Aliakbari S, Kazemi R, Amini AA, Rahmani MR. Hydrocortisone Long-term treatment effect on Immunomodulatory properties of human Adipose-Derived mesenchymal Stromal/Stem cells. J Interferon Cytokine Res. 2022;42(2):72–81. 35171704 10.1089/jir.2021.0120

[167] Durand N, Russell A, Zubair AC. Effect of comedications and endotoxins on mesenchymal stem cell secretomes, migratory and Immunomodulatory capacity. J Clin Med. 2019;8(4):497. 30979082 10.3390/jcm8040497 PMC6517980

[168] Rawat S, Dadhwal V, Mohanty S. Dexamethasone priming enhances stemness and Immunomodulatory property of tissue-specific human mesenchymal stem cells. BMC Dev Biol. 2021;21(1):16. 34736395 10.1186/s12861-021-00246-4 PMC8567134

[169] Genç D, Zibandeh N, Nain E, Arığ Ü, Göker K, Aydıner EK, et al. IFN-γ stimulation of dental follicle mesenchymal stem cells modulates immune response of CD4(+) T lymphocytes in der p1(+) asthmatic patients in vitro. Allergol Immunopathol (Madr). 2019;47(5):467–76. 30826066 10.1016/j.aller.2018.12.005

[170] Deng L, Li H, Su X, Zhang Y, Xu H, Fan L, et al. Chlorzoxazone, a small molecule drug, augments immunosuppressive capacity of mesenchymal stem cells via modulation of FOXO3 phosphorylation. Cell Death Dis. 2020;11(3):158. 32123161 10.1038/s41419-020-2357-8 PMC7052156

[171] Yao M, Chen Z, He X, Long J, Xia X, Li Z, et al. Cross talk between glucose metabolism and immunosuppression in IFN-γ-primed mesenchymal stem cells. Life Sci Alliance. 2022;5(12):e202201493. 36260750 10.26508/lsa.202201493 PMC9463493

[172] Rolandsson Enes S, Hampton TH, Barua J, McKenna DH, Dos Santos CC, Amiel E, et al. Healthy versus inflamed lung environments differentially affect mesenchymal stromal cells. Eur Respir J. 2021;58(4):2004149. 33795318 10.1183/13993003.04149-2020 PMC8543758

[173] Salwierak-Glosna K, Piatek P, Domowicz M, Swiderek-Matysiak M. Effect of multiple sclerosis cerebrospinal fluid and oligodendroglia cell line environment on human wharton’s jelly mesenchymal stem cells secretome. Int J Mol Sci. 2022;23(4):2177. 35216294 10.3390/ijms23042177 PMC8878514

[174] Sayegh S, Atat OE, Diallo K, Rauwel B, Degboe Y, Cavaignac E, et al. Rheumatoid synovial fluids regulate the immunomodulatory potential of adipose-derived mesenchymal stem cells through a TNF/NF-kappaB-dependent mechanism. Front Immunol. 2019;10:1482. 31316519 10.3389/fimmu.2019.01482 PMC6611153

[175] Silva-Carvalho AE, Rodrigues LP, Schiavinato JL, Alborghetti MR, Bettarello G, Simoes BP, et al. GVHD-derived plasma as a priming strategy of mesenchymal stem cells. Stem Cell Res Therapy. 2020;11(1):156. 10.1186/s13287-020-01659-x PMC7164240 32299501

[176] Yousefi F, Arab FL, Jaafari MR, Rastin M, Tabasi N, Hatamipour M, et al. Immunoregulatory, proliferative and anti-oxidant effects of nanocurcuminoids on adipose-derived mesenchymal stem cells. EXCLI J. 2019;18:405–21. 31338010 10.17179/excli2019-1366 PMC6635727

[177] Yousefi F, Arab FL, Rastin M, Tabasi NS, Nikkhah K, Mahmoudi M. Comparative assessment of immunomodulatory, proliferative, and antioxidant activities of Crocin and Crocetin on mesenchymal stem cells. J Cell Biochem. 2021;122(1):29–42. 32951264 10.1002/jcb.29826

[178] Ghasemi F, Khoshmirsafa M, Safari E, Asgari M, Alemrajabi M, Nojehdehi S, et al. Vitamin E and selenium improve mesenchymal stem cell conditioned media immunomodulatory effects. Stem Cell Invest. 2021;8:9. 10.21037/sci-2020-008 PMC8173293 34124232

[179] Park HS, Oh MK, Lee JW, Chae DH, Joo H, Kang JY, et al. Diesel exhaust particles impair therapeutic effect of human wharton’s Jelly-Derived mesenchymal stem cells against experimental colitis through ROS/ERK/cFos signaling pathway. Int J Stem Cells. 2022;15(2):203–16. 34966003 10.15283/ijsc21178 PMC9148831

[180] Uwazie CC, Pirlot BM, Faircloth TU, Patel M, Parr RN, Zastre HM, et al. Effects of atrazine exposure on human bone marrow-derived mesenchymal stromal cells assessed by combinatorial assay matrix. Front Immunol. 2023;14:1214098. 37588595 10.3389/fimmu.2023.1214098 PMC10426140

[181] Bidkhori HR, Farshchian M, Hassanzadeh H, Jafarzadeh Esfehani R, Alsadat Mahmoudian R, Moradi Marjaneh M, et al. Unraveling the effects of DICER1 overexpression on Immune-Related genes expression in mesenchymal Stromal/Stem cells: insights for therapeutic applications. Cell J. 2023;25(10):696–705. 37865878 10.22074/CELLJ.2023.1988987.1221 PMC10591266

[182] Chen Z, Yao MW, Shen ZL, Li SD, Xing W, Guo W, et al. Interferon-gamma and tumor necrosis factor-alpha synergistically enhance the immunosuppressive capacity of human umbilical-cordderived mesenchymal stem cells by increasing PD-L1 expression. World J Stem Cells. 2023;15(8):787–806. 37700823 10.4252/wjsc.v15.i8.787 PMC10494569

[183] Chulpanova DS, Gilazieva ZE, Akhmetzyanova ER, Kletukhina SK, Rizvanov AA, Solovyeva VV. Cytochalasin B-induced membrane vesicles from human mesenchymal stem cells overexpressing TRAIL, PTEN and IFN-beta1 can kill carcinoma cancer cells. Tissue Cell. 2021;73:101664. 34678531 10.1016/j.tice.2021.101664

[184] Dorronsoro A, Lang V, Ferrin I, Fernández-Rueda J, Zabaleta L, Pérez-Ruiz E, et al. Intracellular role of IL-6 in mesenchymal stromal cell immunosuppression and proliferation. Sci Rep. 2020;10(1):21853. 33318571 10.1038/s41598-020-78864-4 PMC7736882

[185] Gómez-Ferrer M, Amaro-Prellezo E, Dorronsoro A, Sánchez-Sánchez R, Vicente Á, Cosín-Roger J, et al. HIF-overexpression and pro-inflammatory priming in human mesenchymal stromal cells improves the healing properties of extracellular vesicles in experimental Crohn’s disease. Int J Mol Sci. 2021;22(20):11269. 34681929 10.3390/ijms222011269 PMC8540690

[186] Gómez-Ferrer M, Villanueva-Badenas E, Sánchez-Sánchez R, Sánchez-López CM, Baquero MC, Sepúlveda P, et al. HIF-1α and pro-inflammatory signaling improves the immunomodulatory activity of MSC-derived extracellular vesicles. Int J Mol Sci. 2021;22(7):3416. 33810359 10.3390/ijms22073416 PMC8036951

[187] Heidari F, Razmkhah M, Razban V, Erfani N. Effects of indoleamine 2, 3-dioxygenase (IDO) Silencing on Immunomodulatory function and cancer-promoting characteristic of adipose-derived mesenchymal stem cells (ASCs). Cell Biol Int. 2021;45(12):2544–56. 34498786 10.1002/cbin.11698

[188] Korchak JA, Delawary M, Huang P, Zhang C, Suda K, Zubair AC. Endothelial nitric oxide synthase-engineered mesenchymal stromal cells induce anti-inflammation in experimental immune models. Cytotherapy. 2022;24(3):262–71. 34836820 10.1016/j.jcyt.2021.10.001

[189] Liu M, Chen J, Huang H, Zeng Y, Feng X, Shi M. Lkb1 is an important regulator of Treg differentiation and proliferation of amniotic mesenchymal stem cells. Biochem Biophys Res Commun. 2020;521(2):434–40. 31672271 10.1016/j.bbrc.2019.09.129

[190] Na T, Zhang K, Yuan BZ. The DLC-1 tumor suppressor is involved in regulating Immunomodulation of human mesenchymal stromal /stem cells through interacting with the Notch1 protein. BMC Cancer. 2020;20(1):1064. 33148199 10.1186/s12885-020-07542-5 PMC7640439

[191] Ortiz-Virumbrales M, Menta R, Pérez LM, Lucchesi O, Mancheño-Corvo P, Avivar-Valderas Á, et al. Human adipose mesenchymal stem cells modulate myeloid cells toward an anti-inflammatory and reparative phenotype: role of IL-6 and PGE2. Stem Cell Res Ther. 2020;11(1):462. 33138862 10.1186/s13287-020-01975-2 PMC7607855

[192] Ozdemir AT, Nalbantsoy A, Ozgul Ozdemir RB, Berdeli A. Effects of 15-lipoxygenase overexpressing adipose tissue mesenchymal stem cells on the Th17 / Treg plasticity. Prostaglandins Other Lipid Mediat. 2022;159:106610. 34963632 10.1016/j.prostaglandins.2021.106610

[193] Pers YM, Bony C, Duroux-Richard I, Bernard L, Maumus M, Assou S, et al. miR-155 contributes to the immunoregulatory function of human mesenchymal stem cells. Front Immunol. 2021;12:624024. 33841404 10.3389/fimmu.2021.624024 PMC8033167

[194] Ramos-Murillo AI, Rodriguez E, Beltran K, Ricaurte C, Camacho B, Salguero G, et al. Efficient non-viral gene modification of mesenchymal stromal cells from umbilical cord wharton’s jelly with polyethylenimine. Pharmaceutics. 2020;12(9):1–19. 10.3390/pharmaceutics12090896 PMC7559368 32971730

[195] Schäfer R, Schwab M, Siegel G, von Ameln-Mayerhofer A, Buadze M, Lourhmati A, et al. Modulating endothelial adhesion and migration impacts stem cell therapies efficacy. EBioMedicine. 2020;60:102987. 32942121 10.1016/j.ebiom.2020.102987 PMC7498853

[196] Selich A, Zimmermann K, Tenspolde M, Dittrich-Breiholz O, von Kaisenberg C, Schambach A, et al. Umbilical cord as a long-term source of activatable mesenchymal stromal cells for Immunomodulation. Stem Cell Res Ther. 2019;10(1):285. 31547865 10.1186/s13287-019-1376-9 PMC6755709

[197] Shi S, Chen S, Liang B, Li Y, Ma Q, Li M, et al. Improved therapeutic consistency and efficacy of CD317 + MSCs through stabilizing TSG6 by PTX3. Stem Cell Res Therapy. 2024;15(1):92. 10.1186/s13287-024-03706-3 PMC10976765 38539221

[198] Yoshii H, Kajiya M, Yoshino M, Morimoto S, Horikoshi S, Tari M, et al. Mechanosignaling YAP/TAZ-TEAD axis regulates the Immunomodulatory properties of mesenchymal stem cells. Stem Cell Reviews Rep. 2024;20(1):347–61. 10.1007/s12015-023-10646-7 37917410

[199] Heidari N, Mohammadi M, Rezaee MA, Amini AA, Fakhari S, Rahmani MR. Up-regulation of CD200/CD200R1 Immunomodulatory axis of allogenic peripheral blood mononuclear cells in a Co-culture with adipose-derived mesenchymal stem cells. Iran J Allergy Asthma Immunol. 2021;20(1):484–96. 33463116 10.18502/ijaai.v19i5.4464

[200] Tran TDX, Pham VQ, Tran NNT, Dang HCN, Tran NTA, Vu NB, et al. Stromal vascular fraction and mesenchymal stem cells from human adipose tissue: a comparison of immune modulation and angiogenic Potential. Adv Exp Med Biol. 2023;Part F1638:47–61. 10.1007/5584_2022_708 35389201

[201] Tawfeek GAE, Esaily HA. A novel function of collagen/PCL nanofiber interaction with MSCs in osteoarthritis is potentiation its Immunomodulatory effect through increased ICAM expression. Transpl Immunol. 2022;73:101625. 35569718 10.1016/j.trim.2022.101625

[202] Pham LH, Vu NB, Van Pham P. The subpopulation of CD105 negative mesenchymal stem cells show strong Immunomodulation capacity compared to CD105 positive mesenchymal stem cells. Biomedical Res Therapy. 2019;6(4):3131–40.

[203] Tesarova L, Jaresova K, Simara P, Koutna I. Umbilical cord-derived mesenchymal stem cells are able to use bFGF treatment and represent a superb tool for immunosuppressive clinical applications. Int J Mol Sci. 2020;21(15):5366. 32731615 10.3390/ijms21155366 PMC7432622

[204] Piede N, Bremm M, Farken A, Pfeffermann LM, Cappel C, Bonig H, et al. Validation of an ICH Q2 compliant flow cytometry-based assay for the assessment of the inhibitory potential of mesenchymal stromal cells on T cell proliferation. Cells. 2023;12(6):850. 36980191 10.3390/cells12060850 PMC10047294

[205] Laroye C, Gauthier M, Antonot H, Decot V, Reppel L, Bensoussan D. Mesenchymal stem/stromal cell production compliant with good manufacturing practice: comparison between bone marrow, the gold standard adult source, and wharton’s jelly, an extraembryonic source. J Clin Med. 2019;8(12):2207. 31847319 10.3390/jcm8122207 PMC6947040

[206] Cruz-Barrera M, Flórez-Zapata N, Lemus-Diaz N, Medina C, Galindo CC, González-Acero LX, et al. Integrated analysis of transcriptome and secretome from umbilical cord mesenchymal stromal cells reveal new mechanisms for the modulation of inflammation and immune activation. Front Immunol. 2020;11:575488. 33117373 10.3389/fimmu.2020.575488 PMC7561386

[207] Piekarska K, Urban-Wójciuk Z, Kurkowiak M, Pelikant-Małecka I, Schumacher A, Sakowska J, et al. Mesenchymal stem cells transfer mitochondria to allogeneic Tregs in an HLA-dependent manner improving their immunosuppressive activity. Nat Commun. 2022;13(1):856. 35165293 10.1038/s41467-022-28338-0 PMC8844425

[208] Koh B, Sulaiman N, Fauzi MB, Law JX, Ng MH, Yuan TL, et al. A Three-Dimensional Xeno-Free culture condition for wharton’s Jelly-Mesenchymal stem cells: the pros and cons. Int J Mol Sci. 2023;24(4):3745. 36835154 10.3390/ijms24043745 PMC9960744

[209] Cerqueira A, Romero-Gavilán F, Helmholz H, Azkargorta M, Elortza F, Gurruchaga M, et al. Proteomic analysis of mesenchymal stem cells and monocyte Co-Cultures exposed to a bioactive Silica-Based Sol-Gel coating. ACS Biomater Sci Eng. 2023;9(6):3306–19. 37202924 10.1021/acsbiomaterials.3c00254 PMC10265575

[210] Di Tinco R, Bertani G, Pisciotta A, Bertoni L, Pignatti E, Maccaferri M, et al. Role of PD-L1 in licensing immunoregulatory function of dental pulp mesenchymal stem cells. Stem Cell Res Ther. 2021;12(1):598. 34863286 10.1186/s13287-021-02664-4 PMC8643194

[211] Baharlooi H, Salehi Z, Moeini MM, Rezaei N, Azimi M. Immunomodulatory potential of human mesenchymal stem cells and their exosomes on multiple sclerosis. Adv Pharm Bull. 2022;12(2):389–97. 35620339 10.34172/apb.2022.038 PMC9106959

[212] Carreras-Planella L, Monguio-Tortajada M, Enric Borras F, Franquesa M. Immunomodulatory effect of MSC on B cells is independent of secreted extracellular vesicles. Front Immunol. 2019;10(JUN):1288. 31244839 10.3389/fimmu.2019.01288 PMC6563675

[213] Mahmoudi M, Taghavi-Farahabadi M, Rezaei N, Hashemi SM. Comparison of the effects of adipose tissue mesenchymal stromal cell-derived exosomes with conditioned media on neutrophil function and apoptosis. Int Immunopharmacol. 2019;74:105689. 31207404 10.1016/j.intimp.2019.105689

[214] Serejo TRT, Silva-Carvalho AE, Braga LDCF, Neves FAR, Pereira EW, Carvalho JL, et al. Assessment of the immunosuppressive potential of INF-gamma licensed adipose mesenchymal stem cells, their secretome and extracellular vesicles. Cells. 2019;8(1):22. 30621275 10.3390/cells8010022 PMC6356584

[215] Taghavi-Farahabadi M, Mahmoudi M, Rezaei N, Hashemi SM. Wharton’s jelly mesenchymal stem cells exosomes and conditioned media increased neutrophil lifespan and phagocytosis capacity. Immunol Invest. 2021;50(8):1042–57. 32777963 10.1080/08820139.2020.1801720

[216] Gonzalez-Pujana A, de Lázaro I, Vining KH, Santos-Vizcaino E, Igartua M, Hernandez RM, et al. 3D encapsulation and inflammatory licensing of mesenchymal stromal cells alter the expression of common reference genes used in real-time RT-qPCR. Biomater Sci. 2020;8(23):6741–53. 33136110 10.1039/d0bm01562h PMC7717608

[217] Herger N, Heggli I, Mengis T, Devan J, Arpesella L, Brunner F, et al. Impacts of priming on distinct immunosuppressive mechanisms of mesenchymal stromal cells under translationally relevant conditions. Stem Cell Res Ther. 2024;15(1):65. 38443999 10.1186/s13287-024-03677-5 PMC10916130

[218] Silva-Carvalho AE, Silva IGMD, Correa JR, Saldanha-Araujo F, Regulatory T-C, Enhancement. Expression of adhesion Molecules, and production of Anti-Inflammatory factors are differentially modulated by Spheroid-Cultured mesenchymal stem cells. Int J Mol Sci. 2022;23(22):14349. 36430835 10.3390/ijms232214349 PMC9695986

[219] Gil-Chinchilla JI, Bueno C, Martínez CM, Ferrández-Múrtula A, García-Hernández AM, Blanquer M, et al. Optimizing cryopreservation conditions for use of fucosylated human mesenchymal stromal cells in anti-inflammatory/immunomodulatory therapeutics. Front Immunol. 2024;15:1385691. 38605955 10.3389/fimmu.2024.1385691 PMC11007032

[220] Silini AR, Papait A, Cargnoni A, Vertua E, Romele P, Bonassi Signoroni P, et al. CM from intact hAM: an easily obtained product with relevant implications for translation in regenerative medicine. Stem Cell Res Therapy. 2021;12(1):540. 10.1186/s13287-021-02607-z PMC8513276 34641958

[221] Kwee BJ, Lam J, Akue A, KuKuruga MA, Zhang K, Gu L, et al. Functional heterogeneity of IFN-γ-licensed mesenchymal stromal cell immunosuppressive capacity on biomaterials. Proc Natl Acad Sci U S A. 2021;118:35. 10.1073/pnas.2105972118 PMC8536328 34446555

[222] Kolliopoulos V, Polanek M, Xu H, Harley B. Inflammatory licensed hMSCs exhibit enhanced Immunomodulatory capacity in a biomaterial mediated manner. ACS Biomaterials Sci Eng. 2023;9(8):4916–28. 10.1021/acsbiomaterials.3c00290 PMC10600978 37390452

[223] Haseli M, Pinzon-Herrera L, Hao X, Wickramasinghe SR, Almodovar J. Novel strategy to enhance human mesenchymal stromal cell immunosuppression: Harnessing Interferon-Gamma presentation in Metal-Organic frameworks embedded on Heparin/Collagen multilayers. Langmuir. 2023;39(46):16472–83. 37944116 10.1021/acs.langmuir.3c02355

[224] Dunn CM, Kameishi S, Parker T, Cho YK, Song SU, Grainger DW, et al. Cellular interactions in cell sheets enhance mesenchymal stromal cell Immunomodulatory properties. Tissue Eng Part A. 2023;29(21–22):594–603. 37847176 10.1089/ten.TEA.2023.0059

[225] Gao S, Jin Y, Ma J, Wang J, Shao Z, Fan T, et al. Preclinical study of human umbilical cord mesenchymal stem cell sheets for the recovery of ischemic heart tissue. Stem Cell Res Ther. 2022;13(1):252. 35690871 10.1186/s13287-022-02919-8 PMC9188245

[226] Hyland M, Mennan C, Wilson E, Clayton A, Kehoe O. Pro-Inflammatory priming of umbilical cord mesenchymal stromal cells alters the protein cargo of their extracellular vesicles. Cells. 2020;9(3):726. 32188006 10.3390/cells9030726 PMC7140705

[227] Li H, Ji XQ, Zhang SM, Bi RH. Hypoxia and inflammatory factor preconditioning enhances the immunosuppressive properties of human umbilical cord mesenchymal stem cells. World J Stem Cells. 2023;15(11):999–1016. 38058960 10.4252/wjsc.v15.i11.999 PMC10696190

[228] Merkhan MM, Shephard MT, Forsyth NR. Physoxia alters human mesenchymal stem cell secretome. J Tissue Eng. 2021;12:20417314211056132. 34733464 10.1177/20417314211056132 PMC8558798

[229] Wedzinska A, Figiel-Dabrowska A, Kozlowska H, Sarnowska A. The effect of Proinflammatory cytokines on the proliferation, migration and secretory activity of mesenchymal stem/stromal cells (Wj-mscs) under 5% o2 and 21% o2 culture conditions. J Clin Med. 2021;10(9):1813. 33919308 10.3390/jcm10091813 PMC8122617

[230] Wu J, Huang QM, Liu Y, Zhou J, Tang WR, Wang XY, et al. Long-term hypoxic hUCMSCs-derived extracellular vesicles alleviates allergic rhinitis through triggering immunotolerance of their VEGF-mediated Inhibition of dendritic cells maturation. Int Immunopharmacol. 2023;124(Pt B):110875. 37742368 10.1016/j.intimp.2023.110875

[231] Khasawneh RR, Abu-El-Rub E, Almahasneh FA, Alzu’bi A, Zegallai HM, Almazari RA, et al. Addressing the impact of high glucose microenvironment on the immunosuppressive characteristics of human mesenchymal stem cells. IUBMB Life. 2024;76(5):286–95. 38014654 10.1002/iub.2796

[232] Herzig MC, Delavan CP, Jensen KJ, Cantu C, Montgomery RK, Christy BA, et al. A streamlined proliferation assay using mixed lymphocytes for evaluation of human mesenchymal stem cell Immunomodulation activity. J Immunol Methods. 2021;488:112915. 33212091 10.1016/j.jim.2020.112915

[233] Marino LS, Nithya TG, Julius A. Lyophilized human platelet lysate as a supplementation in the culture of umbilical cord derived mesenchymal stem cells. Tissue Cell. 2023;82:102092. 37075679 10.1016/j.tice.2023.102092

[234] Mebarki M, Iglicki N, Marigny C, Abadie C, Nicolet C, Churlaud G, et al. Development of a human umbilical cord-derived mesenchymal stromal cell-based advanced therapy medicinal product to treat immune and/or inflammatory diseases. Stem Cell Res Ther. 2021;12(1):571. 34774107 10.1186/s13287-021-02637-7 PMC8590372

[235] Nicotra T, Desnos A, Halimi J, Antonot H, Reppel L, Belmas T, et al. Mesenchymal stem/stromal cell quality control: validation of mixed lymphocyte reaction assay using flow cytometry according to ICH Q2(R1). Stem Cell Res Therapy. 2020;11(1):426. 10.1186/s13287-020-01947-6 PMC7531151 33004063

[236] Ramos YFM, Tertel T, Shaw G, Staubach S, de Almeida RC, Suchiman E, et al. Characterizing the secretome of licensed hiPSC-derived MSCs. Stem Cell Res Ther. 2022;13(1):434. 36056373 10.1186/s13287-022-03117-2 PMC9438242

[237] Zhao Y, Song W, Yuan Z, Li M, Wang G, Wang L, et al. Exosome derived from human umbilical cord mesenchymal cell exerts Immunomodulatory effects on B cells from SLE patients. J Immunol Res. 2023;2023:3177584. 37215068 10.1155/2023/3177584 PMC10198761

[238] Behm C, Blufstein A, Gahn J, Kubin B, Moritz A, Rausch-Fan X, et al. Continuing effect of cytokines and toll-like receptor agonists on indoleamine-2,3-dioxygenase-1 in human periodontal ligament stem/stromal cells. Cells. 2020;9(12):1–16. 10.3390/cells9122696 PMC7765527 33339125

[239] Behm C, Blufstein A, Gahn J, Kubin B, Nemec M, Moritz A, et al. 1,25(Oh)2d3 differently affects Immunomodulatory activities of mesenchymal stem cells depending on the presence of TNF-alpha, IL-1beta and IFN-gamma. J Clin Med. 2019;8(12):2211. 31847340 10.3390/jcm8122211 PMC6947512

[240] Behm C, Blufstein A, Gahn J, Moritz A, Rausch-Fan X, Andrukhov O. 25-hydroxyvitamin D(3) generates Immunomodulatory plasticity in human periodontal ligament-derived mesenchymal stromal cells that is inflammatory context-dependent. Front Immunol. 2023;14:1100041. 36761739 10.3389/fimmu.2023.1100041 PMC9902380

[241] Bowles AC, Kouroupis D, Willman MA, Perucca Orfei C, Agarwal A, Correa D. Signature quality attributes of CD146(+) mesenchymal stem/stromal cells correlate with high therapeutic and secretory potency. Stem Cells. 2020;38(8):1034–49. 32379908 10.1002/stem.3196

[242] Inostroza C, Vega-Letter AM, Brizuela C, Castrillón L, Saint Jean N, Duran CM, et al. Mesenchymal stem cells derived from human inflamed dental pulp exhibit impaired Immunomodulatory capacity in vitro. J Endod. 2020;46(8):1091–e82. 32422164 10.1016/j.joen.2020.05.003

[243] Jang SG, Lee J, Hong SM, Kwok SK, Cho ML, Park SH. Metformin enhances the Immunomodulatory potential of adipose-derived mesenchymal stem cells through STAT1 in an animal model of lupus. Rheumatol (United Kingdom). 2020;59(6):1426–38. 10.1093/rheumatology/kez631 31904843

[244] Kouroupis D, Bowles AC, Greif DN, Leñero C, Best TM, Kaplan LD, et al. Regulatory-compliant conditions during cell product manufacturing enhance in vitro Immunomodulatory properties of infrapatellar fat pad-derived mesenchymal stem/stromal cells. Cytotherapy. 2020;22(11):677–89. 32723596 10.1016/j.jcyt.2020.06.007

[245] Li C, Huang J, Zhu H, Shi Q, Li D, Ju X. Pyridoxal-5’-phosphate promotes immunomodulatory function of adipose-derived mesenchymal stem cells through indoleamine 2,3-dioxygenase-1 and TLR4/NF-kappaB pathway. Stem Cells Int. 2019;2019:3121246. 31885603 10.1155/2019/3121246 PMC6899265

[246] Maughon TS, Shen X, Huang D, Michael AOA, Shockey WA, Andrews SH, et al. Metabolomics and cytokine profiling of mesenchymal stromal cells identify markers predictive of T-cell suppression. Cytotherapy. 2022;24(2):137–48. 34696960 10.1016/j.jcyt.2021.08.002

[247] Queckbörner S, Syk Lundberg E, Gemzell-Danielsson K, Davies LC. Endometrial stromal cells exhibit a distinct phenotypic and Immunomodulatory profile. Stem Cell Res Ther. 2020;11(1):15. 31907034 10.1186/s13287-019-1496-2 PMC6945659

[248] Rosado-Galindo H, Domenech M. Substrate topographies modulate the secretory activity of human bone marrow mesenchymal stem cells. Stem Cell Res Ther. 2023;14(1):208. 37605275 10.1186/s13287-023-03450-0 PMC10441765

[249] Schrodt MV, Behan-Bush RM, Liszewski JN, Humpal-Pash ME, Boland LK, Scroggins SM, et al. Efferocytosis of viable versus heat-inactivated MSC induces human monocytes to distinct immunosuppressive phenotypes. Stem Cell Res Ther. 2023;14(1):206. 37592321 10.1186/s13287-023-03443-z PMC10433682

[250] Van Grouw A, Colonna MB, Maughon TS, Shen X, Larey AM, Moore SG, et al. Development of a robust consensus modeling approach for identifying cellular and media metabolites predictive of mesenchymal stromal cell potency. Stem Cells. 2023;41(8):792–808. 37279550 10.1093/stmcls/sxad039 PMC10427967

[251] Grinnemo KH, Löfling M, Nathanson L, Baumgartner R, Ketelhuth DFJ, Beljanski V, et al. Immunomodulatory effects of interferon-γ on human fetal cardiac mesenchymal stromal cells. Stem Cell Res Ther. 2019;10(1):371. 31801632 10.1186/s13287-019-1489-1 PMC6894330

[252] Jeske SS, Theodoraki MN, Boelke E, Laban S, Brunner C, Rotter N, et al. Adenosine production in mesenchymal stromal cells in relation to their developmental status. HNO. 2020;68(2):87–93. 31915882 10.1007/s00106-019-00805-z

[253] Yan F, Liu O, Zhang H, Zhou Y, Zhou D, Zhou Z, et al. Human dental pulp stem cells regulate allogeneic NK cells’ function via induction of anti-inflammatory purinergic signalling in activated NK cells. Cell Prolif. 2019;52(3):e12595. 30953394 10.1111/cpr.12595 PMC6536423

[254] Holopainen M, Impola U, Lehenkari P, Laitinen S, Kerkelä E. Human mesenchymal stromal cell secretome promotes the immunoregulatory phenotype and phagocytosis activity in human macrophages. Cells. 2020;9(9):2142. 32972000 10.3390/cells9092142 PMC7564172

[255] Maqbool M, Algraittee SJR, Boroojerdi MH, Sarmadi VH, John CM, Vidyadaran S, et al. Human mesenchymal stem cells inhibit the differentiation and effector functions of monocytes. Innate Immun. 2019;26(5):424–34. 10.1177/1753425919899132 PMC7903531 32635840

[256] Tan Y, Salkhordeh M, Wang JP, McRae A, Souza-Moreira L, McIntyre L, et al. Thawed mesenchymal stem cell product shows comparable Immunomodulatory potency to cultured cells in vitro and in polymicrobial septic animals. Sci Rep. 2019;9(1):18078. 31792313 10.1038/s41598-019-54462-x PMC6889371

[257] Gomzikova MO, Kletukhina SK, Kurbangaleeva SV, Neustroeva OA, Vasileva OS, Garanina EE, et al. Mesenchymal stem cell derived biocompatible membrane vesicles demonstrate Immunomodulatory activity inhibiting activation and proliferation of human mononuclear cells. Pharmaceutics. 2020;12(6):1–15. 10.3390/pharmaceutics12060577 PMC7356506 32585863

[258] Taghavi-Farahabadi M, Mahmoudi M, Hashemi SM, Rezaei N. Evaluation of the effects of mesenchymal stem cells on neutrophils isolated from severe congenital neutropenia patients. Int Immunopharmacol. 2020;83:106463. 32251962 10.1016/j.intimp.2020.106463

[259] Guo L, Lai P, Wang Y, Huang T, Chen X, Luo C, et al. Extracellular vesicles from mesenchymal stem cells prevent contact hypersensitivity through the suppression of Tc1 and Th1 cells and expansion of regulatory T cells. Int Immunopharmacol. 2019;74:105663. 31200338 10.1016/j.intimp.2019.05.048

[260] da Silva TB, Rendra E, David CAW, Bieback K, Cross MJ, Wilm B, et al. Umbilical cord mesenchymal stromal cell-derived extracellular vesicles lack the potency to Immunomodulate human monocyte-derived macrophages in vitro. Biomed Pharmacotherapy. 2023;167:115624. 10.1016/j.biopha.2023.115624 37783151

[261] Ma D, Wu Z, Zhao X, Zhu X, An Q, Wang Y, et al. Immunomodulatory effects of umbilical mesenchymal stem cell-derived exosomes on CD4(+) T cells in patients with primary Sjögren’s syndrome. Inflammopharmacology. 2023;31(4):1823–38. 37012581 10.1007/s10787-023-01189-x PMC10352432

[262] Blufstein A, Behm C, Kubin B, Gahn J, Moritz A, Rausch-Fan X, et al. Anti-apoptotic effects of human gingival mesenchymal stromal cells on polymorphonuclear leucocytes. Oral Dis. 2022;28(3):777–85. 33386669 10.1111/odi.13768 PMC9290793

[263] Herzig MC, Christy BA, Montgomery RK, Cantu-Garza C, Barrera GD, Lee JH, et al. Short-term assays for mesenchymal stromal cell immunosuppression of T-lymphocytes. Front Immunol. 2023;14:1225047. 37822938 10.3389/fimmu.2023.1225047 PMC10562633

[264] Boland LK, Burand AJ, Boyt DT, Dobroski H, Di L, Liszewski JN, et al. Nature vs. nurture: defining the effects of mesenchymal stromal cell isolation and culture conditions on resiliency to palmitate challenge. Front Immunol. 2019;10:1080. 31134100 10.3389/fimmu.2019.01080 PMC6523025

[265] Rivera-Cruz CM, Figueiredo ML. Evaluation of human adipose-derived mesenchymal stromal cell Toll-like receptor priming and effects on interaction with prostate cancer cells. Cytotherapy. 2023;25(1):33–45. 36257875 10.1016/j.jcyt.2022.09.009

[266] Kim JE, Lee YJ, Lee KJ, Park SH, Kang H. Ex vivo treatment with allogenic mesenchymal stem cells of a healthy donor on peripheral blood mononuclear cells of patients with severe alopecia areata: targeting dysregulated T cells and the acquisition of immunotolerance. Int J Mol Sci. 2022;23(21):13228. 36362015 10.3390/ijms232113228 PMC9655710

[267] Grau-Vorster M, Rodriguez L, Del Mazo-Barbara A, Mirabel C, Blanco M, Codinach M, et al. Compliance with good manufacturing practice in the assessment of Immunomodulation potential of clinical grade multipotent mesenchymal stromal cells derived from wharton’s jelly. Cells. 2019;8(5):484. 31117301 10.3390/cells8050484 PMC6562958

[268] Jia Y, Wang A, Zhao B, Wang C, Su R, Zhang B, et al. An optimized method for obtaining clinical-grade specific cell subpopulations from human umbilical cord-derived mesenchymal stem cells. Cell Prolif. 2022;55(10):e13300. 35768999 10.1111/cpr.13300 PMC9528761

[269] Thej C, Balasubramanian S, Rengasamy M, Walvekar A, Swamynathan P, Raj SS, et al. Human bone marrow-derived, pooled, allogeneic mesenchymal stromal cells manufactured from multiple donors at different times show comparable biological functions in vitro, and in vivo to repair limb ischemia. Stem Cell Res Ther. 2021;12(1):279. 33971964 10.1186/s13287-021-02330-9 PMC8108338

[270] Todtenhaupt P, Franken LA, Groene SG, van Hoolwerff M, van der Meeren LE, van Klink JMM, et al. A robust and standardized method to isolate and expand mesenchymal stromal cells from human umbilical cord. Cytotherapy. 2023;25(10):1057–68. 37516948 10.1016/j.jcyt.2023.07.004

[271] Torrents S, Del Moral AE, Codinach M, Rodríguez L, Querol S, Vives J. Optimized reagents for immunopotency assays on mesenchymal stromal cells for clinical use. Immunol Res. 2023;71(5):725–34. 37120479 10.1007/s12026-023-09385-1 PMC10148700

[272] Renesme L, Cobey KD, Lalu MM, Bubela T, Chinnadurai R, De Vos J, et al. Delphi-driven consensus definition for mesenchymal stromal cells and clinical reporting guidelines for mesenchymal stromal cell–based therapeutics. Cytotherapy. 2025;27(2):146–68. 39580717 10.1016/j.jcyt.2024.10.008 PMC12931451

[273] European Medicines Agency. ICH Q2(R2) validation of analytical procedures—scientific guideline. EMA/CHMP/ICH/82072/2006. EMA/CHMP/ICH/82072/20062024.

[274] U.S. Food and Drug Administration. Guidance for industry—potency tests for cellular and gene therapy products. In: Research CfBEa, editor. 2011.

[275] Azaryan E, Mortazavi-Derazkola S, Alemzadeh E, Emadian Razavi F, Yousefi M, Hanafi-Bojd MY, et al. Effects of hydroxyapatite nanorods prepared through elaeagnus angustifolia extract on modulating immunomodulatory/dentin-pulp regeneration genes in DPSCs. Odontology. 2023;111(2):461–73. 36350427 10.1007/s10266-022-00761-1

[276] Bulati M, Miceli V, Gallo A, Amico G, Carcione C, Pampalone M, et al. The Immunomodulatory properties of the human Amnion-Derived mesenchymal Stromal/Stem cells are induced by INF-γ produced by activated lymphomonocytes and are mediated by Cell-To-Cell contact and soluble factors. Front Immunol. 2020;11:54. 32117234 10.3389/fimmu.2020.00054 PMC7028706

[277] Cheng A, Choi D, Lora M, Shum-Tim D, Rak J, Colmegna I. Human multipotent mesenchymal stromal cells cytokine priming promotes RAB27B-regulated secretion of small extracellular vesicles with Immunomodulatory cargo. Stem Cell Res Ther. 2020;11(1):539. 33317598 10.1186/s13287-020-02050-6 PMC7734842

[278] Contreras-Lopez R, Elizondo-Vega R, Luque-Campos N, Torres MJ, Pradenas C, Tejedor G, et al. The ATP synthase Inhibition induces an AMPK-dependent glycolytic switch of mesenchymal stem cells that enhances their immunotherapeutic potential. Theranostics. 2021;11(1):445–60. 33391485 10.7150/thno.51631 PMC7681096

[279] de Pedro M, Gómez-Serrano M, Marinaro F, López E, Pulido M, Preußer C, et al. IFN-Gamma and TNF-Alpha as a priming strategy to enhance the Immunomodulatory capacity of secretomes from menstrual blood-derived stromal cells. Int J Mol Sci. 2021;22(22):12177. 34830067 10.3390/ijms222212177 PMC8618369

[280] Gonzalez-Cubero E, Gonzalez-Fernandez ML, Olivera ER, Villar-Suarez V. Extracellular vesicle and soluble fractions of adipose tissue-derived mesenchymal stem cells secretome induce inflammatory cytokines modulation in an in vitro model of discogenic pain. Spine J. 2022;22(7):1222–34. 35121152 10.1016/j.spinee.2022.01.012

[281] Kim SH, Das A, Choi HI, Kim KH, Chai JC, Choi MR, et al. Forkhead box O1 (FOXO1) controls the migratory response of Toll-like receptor (TLR3)-stimulated human mesenchymal stromal cells. J Biol Chem. 2019;294(21):8424–37. 30944148 10.1074/jbc.RA119.008673 PMC6544856

[282] Leñero C, Bowles AC, Correa D, Kouroupis D. Characterization and response to inflammatory stimulation of human endometrial-derived mesenchymal stem/stromal cells. Cytotherapy. 2022;24(2):124–36. 34465515 10.1016/j.jcyt.2021.07.005

[283] Szucs D, Miklos V, Monostori T, Guba M, Kun-Varga A, Poliska S, et al. Effect of inflammatory microenvironment on the regenerative capacity of Adipose-Derived mesenchymal stem cells. Cells. 2023;12(15):1966. 37566046 10.3390/cells12151966 PMC10416993

[284] Vereb Z, Mazlo A, Szabo A, Poliska S, Kiss A, Litauszky K, et al. Vessel wall-derived mesenchymal stromal cells share similar differentiation potential and immunomodulatory properties with bone marrow-derived stromal cells. Stem Cells Int. 2020;2020:8847038. 33144864 10.1155/2020/8847038 PMC7596426

[285] Wan Z, Chen YF, Pan Q, Wang Y, Yuan S, Chin HY, et al. Single-cell transcriptome analysis reveals the effectiveness of cytokine priming irrespective of heterogeneity in mesenchymal stromal cells. Cytotherapy. 2023;25(11):1155–66. 37715776 10.1016/j.jcyt.2023.08.006

[286] Andrews SH, Klinker MW, Bauer SR, Marklein RA. Morphological landscapes from high content imaging reveal cytokine priming strategies that enhance mesenchymal stromal cell immunosuppression. Biotechnol Bioeng. 2022;119(2):361–75. 34716713 10.1002/bit.27974

[287] Dubus M, Varin J, Papa S, Chevrier J, Quiles F, Francius G, et al. Bone marrow mesenchymal stem cells offer an immune-privileged niche to Cutibacterium acnes in case of implant-associated osteomyelitis. Acta Biomater. 2022;137:305–15. 34678484 10.1016/j.actbio.2021.10.026

[288] Khalil F, Alwan A, Ralph P, Soliman S, Abdelrahim EA, Abdelhafez EA, et al. Effect of alginate microbead encapsulation of placental mesenchymal stem cells on their Immunomodulatory function. Ann Biomed Eng. 2022;50(3):291–302. 35072884 10.1007/s10439-022-02920-5

[289] McKinnirey F, Herbert B, Vesey G, McCracken S. Immune modulation via adipose derived mesenchymal stem cells is driven by donor sex in vitro. Sci Rep. 2021;11(1):12454. 34127731 10.1038/s41598-021-91870-4 PMC8203671

[290] Mendt M, Daher M, Basar R, Shanley M, Kumar B, Wei Inng FL, et al. Metabolic reprogramming of GMP grade cord tissue derived mesenchymal stem cells enhances their suppressive potential in GVHD. Front Immunol. 2021;12:631353. 34017325 10.3389/fimmu.2021.631353 PMC8130860

[291] Ruhl T, Corsten C, Beier JP, Kim BS. The immunosuppressive effect of the endocannabinoid system on the inflammatory phenotypes of macrophages and mesenchymal stromal cells: a comparative study. Pharmacol Rep. 2021;73(1):143–53. 33026642 10.1007/s43440-020-00166-3

[292] Soleymani-Goloujeh M, Shekari F, Hassani SN, Hajizadeh-Saffar E. Preconditioning with IFN-gamma and LPS improves the Immunomodulatory potential of bone marrow-derived clonal mesenchymal stem cells. Health Biotechnol Biopharma. 2023;7(2):35–51.

[293] Song J, Ma Q, Li Y, Wang X, Chen S, Liang B, et al. CD317(+) MSCs expanded with chemically defined media have enhanced immunological anti-inflammatory activities. Stem Cell Res Ther. 2024;15(1):2. 38169422 10.1186/s13287-023-03618-8 PMC10763464

[294] Spohn G, Witte AS, Kretschmer A, Seifried E, Schafer R. More human BM-MSC. with similar subpopulation composition and functional characteristics can be produced with a GMP-compatible fabric filter system compared to density gradient technique. Front Cell Dev Biol. 2021;9:638798. 33869188 10.3389/fcell.2021.638798 PMC8044851

[295] Hu Z, Li D, Wu S, Pei K, Fu Z, Yang Y, et al. Unveiling the functional heterogeneity of cytokine-primed human umbilical cord mesenchymal stem cells through single-cell RNA sequencing. Cell Bioscience. 2024;14(1):40. 38532459 10.1186/s13578-024-01219-3 PMC10964690

[296] Sun C, Zhang K, Yue J, Meng S, Zhang X. Deconstructing transcriptional variations and their effects on Immunomodulatory function among human mesenchymal stromal cells. Stem Cell Res Ther. 2021;12(1):53. 33422149 10.1186/s13287-020-02121-8 PMC7796611

[297] Zhou Z, Wu X, Chen T, Zhang B, Li W, Zhou M, et al. Restoration of functional endometrium in an intrauterine adhesion rat model with endometrial stromal cells transplantation. Stem Cell Res Ther. 2024;15(1):181. 38902788 10.1186/s13287-024-03788-z PMC11191336

[298] Han X, Na T, Wu T, Yuan BZ. Human lung epithelial BEAS-2B cells exhibit characteristics of mesenchymal stem cells. PLoS ONE. 2020;15(1):e0227174. 31900469 10.1371/journal.pone.0227174 PMC6941928

[299] Zielniok K, Burdzinska A, Kaleta B, Zagozdzon R, Paczek L. Vadadustat, a HIF Prolyl hydroxylase Inhibitor, improves Immunomodulatory properties of human mesenchymal stromal cells. Cells. 2020;9(11):2396. 33139632 10.3390/cells9112396 PMC7693843

[300] Dolp R, Eylert G, Auger C, Aijaz A, Chen YA, Amini-Nik S, et al. Biological characteristics of stem cells derived from burned skin-a comparative study with umbilical cord stem cells. Stem Cell Res Ther. 2021;12(1):137. 33597003 10.1186/s13287-021-02140-z PMC7888080

[301] Jung N, Kong T, Yu Y, Park H, Lee E, Yoo S, et al. Immunomodulatory effect of epidermal growth factor secreted by human umbilical cord Blood-Derived mesenchymal stem cells on atopic dermatitis. Int J Stem Cells. 2022;15(1):1–13. 35220283 10.15283/ijsc21173 PMC9396020

[302] Faircloth TU, Temple S, Parr RN, Tucker AB, Rajan D, Hematti P, et al. Vascular endothelial growth factor secretion and immunosuppression are distinct potency mechanisms of human bone marrow mesenchymal stromal cells. Stem Cells. 2024;42(8):736–51. 38826008 10.1093/stmcls/sxae040

[303] Junior AL, Pinheiro CCG, Tanikawa DYS, Ferreira JRM, Amano MT, Bueno DF. Mesenchymal stem cells from human exfoliated deciduous teeth and the orbicularis oris muscle: how do they behave when exposed to a proinflammatory stimulus? Stem Cells Int. 2020;2020:3670412. 32184831 10.1155/2020/3670412 PMC7060870

[304] Liu N, Cheng Y, Wang D, Guan H, Chen D, Zeng J, et al. Tissue-specific populations from amniotic fluid-derived mesenchymal stem cells manifest variant in vitro and in vivo properties. Hum Cell. 2024;37(2):408–19. 38085460 10.1007/s13577-023-01008-z PMC10891244

[305] He YB, Zhang L, Zhou LL, Chen YM, Lu JH, Chen J, et al. Effect of human follicle-stimulating hormone on Immunomodulatory function of decidual mesenchymal stem cells by reducing interleukin-6 levels. J Ovarian Res. 2022;15(1):60. 35562770 10.1186/s13048-022-00993-3 PMC9102716

[306] Satani N, Zhang X, Giridhar K, Wewior N, Cai C, Aronowski J, et al. A combination of atorvastatin and aspirin enhances the pro-regenerative interactions of marrow stromal cells and stroke-derived monocytes in vitro. Front Pharmacol. 2021;12:589418. 33959001 10.3389/fphar.2021.589418 PMC8093790

[307] Blazquez R, Sanchez-Margallo FM, Reinecke J, Alvarez V, Lopez E, Marinaro F, et al. Conditioned serum enhances the chondrogenic and Immunomodulatory behavior of mesenchymal stem cells. Front Pharmacol. 2019;10(JUN):699. 31316380 10.3389/fphar.2019.00699 PMC6609570

[308] Chang SH, Kim HJ, Park CG. Allogeneic ADSCs induce the production of alloreactive memory-CD8 T cells through HLA-ABC antigens. Cells. 2020;9(5):1246. 32443511 10.3390/cells9051246 PMC7290988

[309] Cho WJ, Mittal SK, Chauhan SK. Mesenchymal stromal cells suppress T-Cell-Mediated Delayed-Type hypersensitivity via ALCAM-CD6 interaction. Stem Cells Transl Med. 2023;12(4):221–33. 36972356 10.1093/stcltm/szad012 PMC10108723

[310] Dal Collo G, Adamo A, Gatti A, Tamellini E, Bazzoni R, Takam Kamga P, et al. Functional dosing of mesenchymal stromal cell-derived extracellular vesicles for the prevention of acute graft-versus-host-disease. Stem Cells. 2020;38(5):698–711. 32064745 10.1002/stem.3160

[311] Deng X, Zhang S, Qing Q, Wang P, Ma H, Ma Q, et al. Distinct biological characteristics of mesenchymal stem cells separated from different components of human placenta. Biochem Biophys Rep. 2024;39:101739. 38974020 10.1016/j.bbrep.2024.101739 PMC11225169

[312] Fitzgerald JC, Shaw G, Murphy JM, Barry F. Media matters: culture medium-dependent hypervariable phenotype of mesenchymal stromal cells. Stem Cell Res Ther. 2023;14(1):363. 38087388 10.1186/s13287-023-03589-w PMC10717324

[313] Hejretová L, Čedíková M, Dolejšová M, Vlas T, Jindra P, Lysák D, et al. Comparison of the Immunomodulatory effect of single MSC batches versus pooled MSC products. Cell Tissue Bank. 2020;21(1):119–29. 31863261 10.1007/s10561-019-09805-3

[314] Jakob M, Hambrecht M, Spiegel JL, Kitz J, Canis M, Dressel R, et al. Pluripotent stem cell-derived mesenchymal stem cells show comparable functionality to their autologous origin. Cells. 2020;10(1):33. 33379312 10.3390/cells10010033 PMC7823915

[315] Liao Y, Fu Z, Huang Y, Wu S, Wang Z, Ye S, et al. Interleukin-18-primed human umbilical cord-mesenchymal stem cells achieve superior therapeutic efficacy for severe viral pneumonia via enhancing T-cell immunosuppression. Cell Death Dis. 2023;14(1):66. 36707501 10.1038/s41419-023-05597-3 PMC9883134

[316] Marklein RA, Klinker MW, Drake KA, Polikowsky HG, Lessey-Morillon EC, Bauer SR. Morphological profiling using machine learning reveals emergent subpopulations of interferon-gamma-stimulated mesenchymal stromal cells that predict immunosuppression. Cytotherapy. 2019;21(1):17–31. 30503100 10.1016/j.jcyt.2018.10.008

[317] Montalban-Hernandez K, Casado-Sanchez C, Avendano-Ortiz J, Casalvilla-Duenas JC, Bonel-Perez GC, Prado-Montero J, et al. Fused cells between Human-Adipose-Derived mesenchymal stem cells and monocytes keep stemness properties and acquire high mobility. Int J Mol Sci. 2022;23(17):9672. 36077075 10.3390/ijms23179672 PMC9456160

[318] Psaroudis RT, Singh U, Lora M, Jeon P, Boursiquot A, Stochaj U, et al. CD26 is a senescence marker associated with reduced immunopotency of human adipose tissue-derived multipotent mesenchymal stromal cells. Stem Cell Res Ther. 2022;13(1):358. 35883188 10.1186/s13287-022-03026-4 PMC9327293

[319] Sengun E, Wolfs TGAM, van Bruggen VLE, van Cranenbroek B, Simonetti ER, Ophelders D, et al. Umbilical cord-mesenchymal stem cells induce a memory phenotype in CD4 + T cells. Front Immunol. 2023;14:1128359. 37409122 10.3389/fimmu.2023.1128359 PMC10318901

[320] Sun Y, Wang TE, Hu Q, Zhang W, Zeng Y, Lai X, et al. Systematic comparation of the biological and transcriptomic landscapes of human amniotic mesenchymal stem cells under serum-containing and serum-free conditions. Stem Cell Res Therapy. 2022;13(1):490. 10.1186/s13287-022-03179-2 PMC9530421 36195964

[321] Vázquez A, Fernández-Sevilla LM, Jiménez E, Pérez-Cabrera D, Yañez R, Subiza JL, et al. Involvement of mesenchymal stem cells in oral mucosal bacterial immunotherapy. Front Immunol. 2020;11:567391. 33329530 10.3389/fimmu.2020.567391 PMC7711618

[322] Yang K, Lu R, Lu J, Fan S, Zhang Q, Lou Z, et al. Phenotypic and functional characterizations of mesenchymal stem/stromal cells isolated from human cranial bone marrow. Front NeuroSci. 2022;16:909256. 35747205 10.3389/fnins.2022.909256 PMC9209782

[323] Yoo M, Cho S, Shin S, Kim JM, Park HG, Hwang YK, et al. Therapeutic effect of IL1β priming tonsil Derived-Mesenchymal stem cells in osteoporosis. Tissue Eng Regen Med. 2021;18(5):851–62. 34115339 10.1007/s13770-021-00350-3 PMC8440756

[324] Yu Y, Valderrama AV, Han Z, Uzan G, Naserian S, Oberlin E. Human fetal liver MSCs are more effective than adult bone marrow MSCs for their immunosuppressive, immunomodulatory, and Foxp3(+) T reg induction capacity. Stem Cell Res Ther. 2021;12(1):138. 33597011 10.1186/s13287-021-02176-1 PMC7888159

[325] Heo JS, Choi Y, Kim HO, Matta C. Adipose-derived mesenchymal stem cells promote M2 macrophage phenotype through exosomes. Stem Cells Int. 2019;2019:7921760. 31781246 10.1155/2019/7921760 PMC6875419

[326] Yu C, Tang W, Lu R, Tao Y, Ren T, Gao Y. Human Adipose-derived mesenchymal stem cells promote lymphocyte apoptosis and alleviate atherosclerosis via miR-125b-1-3p/BCL11B signal axis. Annals Palliat Med. 2021;10(2):2123–33. 10.21037/apm-21-49 33725769

[327] Bacic A, Prgomet D, Janjanin S. Tonsil-derived mesenchymal stem cells exert immunosuppressive effects on T cells. Croatian Med J. 2019;60(1):12–9. 10.3325/cmj.2019.60.12 PMC6406067 30825273

[328] Thamm K, Möbus K, Towers R, Baertschi S, Wetzel R, Wobus M, et al. A chemically defined biomimetic surface for enhanced isolation efficiency of high-quality human mesenchymal stromal cells under xenogeneic/serum-free conditions. Cytotherapy. 2022;24(10):1049–59. 35931601 10.1016/j.jcyt.2022.06.003

[329] Viau S, Lagrange A, Chabrand L, Lorant J, Charrier M, Rouger K, et al. A highly standardized and characterized human platelet lysate for efficient and reproducible expansion of human bone marrow mesenchymal stromal cells. Cytotherapy. 2019;21(7):738–54. 31133491 10.1016/j.jcyt.2019.04.053

[330] Bakreen A, Juntunen M, Dunlop Y, Ugidos IF, Malm T, Miettinen S, et al. Additive behavioral improvement after combined cell therapy and rehabilitation despite long-term microglia presence in stroke rats. Int J Mol Sci. 2021;22(4):1–17. 10.3390/ijms22041512 PMC7913568 33546370

[331] Allen A, Vaninov N, Li M, Nguyen S, Singh M, Igo P, et al. Mesenchymal stromal cell bioreactor for ex vivo reprogramming of human immune cells. Sci Rep. 2020;10(1):10142. 32576889 10.1038/s41598-020-67039-w PMC7311545

[332] Kordelas L, Schwich E, Dittrich R, Horn PA, Beelen DW, Borger V, et al. Individual immune-modulatory capabilities of MSC-derived extracellular vesicle (EV) preparations and recipient-dependent responsiveness. Int J Mol Sci. 2019;20(7):1642. 30987036 10.3390/ijms20071642 PMC6479947

[333] Ma C, Feng Y, Yang L, Wang S, Sun X, Tai S, et al. In vitro Immunomodulatory effects of human umbilical Cord-Derived mesenchymal stem cells on peripheral blood cells from warm autoimmune hemolytic anemia patients. Acta Haematol. 2022;145(1):63–71. 34284381 10.1159/000506759

[334] Ma D, Xu K, Zhang G, Liu Y, Gao J, Tian M, et al. Immunomodulatory effect of human umbilical cord mesenchymal stem cells on T lymphocytes in rheumatoid arthritis. Int Immunopharmacol. 2019;74:105687. 31295689 10.1016/j.intimp.2019.105687

[335] Zhu XY, Klomjit N, Conley SM, Ostlie MM, Jordan KL, Lerman A, et al. Impaired Immunomodulatory capacity in adipose tissue-derived mesenchymal stem/stromal cells isolated from obese patients. J Cell Mol Med. 2021;25(18):9051–9. 34418300 10.1111/jcmm.16869 PMC8435432

[336] Zibandeh N, Genc D, Inanc N, Direskeneli H, Akkoc T. IFN-gamma stimulated dental follicle mesenchymal stem cells regulate activated lymphocyte response in rheumatoid arthritis patients in vitro. Turk J Med Sci. 2019;49(6):1779–88. 31655532 10.3906/sag-1812-152 PMC7520073

[337] Caplan HW, Prabhakara KS, Toledano Furman NE, Zorofchian S, Martin C, Xue H, et al. Human-derived Treg and MSC combination therapy May augment immunosuppressive potency in vitro, but did not improve blood brain barrier integrity in an experimental rat traumatic brain injury model. PLoS ONE. 2021;16(5):e0251601. 34038436 10.1371/journal.pone.0251601 PMC8153465

[338] Najar M, Rahmani S, Faour WH, Alsabri SG, Lombard CA, Fayyad-Kazan H, et al. Umbilical cord mesenchymal Stromal/Stem cells and their interplay with Th-17 cell response pathway. Cells. 2024;13(2):169. 38247860 10.3390/cells13020169 PMC10814115

[339] Yao G, Qi J, Liang J, Shi B, Chen W, Li W, et al. Mesenchymal stem cell transplantation alleviates experimental Sjögren’s syndrome through IFN-β/IL-27 signaling axis. Theranostics. 2019;9(26):8253–65. 31754394 10.7150/thno.37351 PMC6857067

[340] Wang JF, Yang XX, Zhang J, Zheng Y, Zhang FQ, Shi XF, et al. Immunomodulation of adipose-derived mesenchymal stem cells on peripheral blood mononuclear cells in colorectal cancer patients with COVID-19. World J Gastrointest Oncol. 2024;16(5):2113–22. 38764823 10.4251/wjgo.v16.i5.2113 PMC11099452

[341] Do JS, Zwick D, Kenyon JD, Zhong F, Askew D, Huang AY, et al. Mesenchymal stromal cell mitochondrial transfer to human induced T-regulatory cells mediates FOXP3 stability. Sci Rep. 2021;11(1):10676. 34021231 10.1038/s41598-021-90115-8 PMC8140113

[342] Ragni E, Perucca Orfei C, De Luca P, Viganò M, Colombini A, Lugano G, et al. miR-22-5p and miR-29a-5p are reliable reference genes for analyzing extracellular Vesicle-Associated MiRNAs in Adipose-Derived mesenchymal stem cells and are stable under inflammatory priming mimicking osteoarthritis condition. Stem Cell Rev Rep. 2019;15(5):743–54. 31161551 10.1007/s12015-019-09899-y

[343] Vaka R, Khan S, Ye B, Risha Y, Parent S, Courtman D, et al. Direct comparison of different therapeutic cell types susceptibility to inflammatory cytokines associated with COVID-19 acute lung injury. Stem Cell Res Ther. 2022;13(1):20. 35033181 10.1186/s13287-021-02699-7 PMC8760881

[344] Kouroupis D, Kaplan LD, Huard J, Best TM. CD10-bound human mesenchymal stem/stromal cell-derived small extracellular vesicles possess Immunomodulatory cargo and maintain cartilage homeostasis under inflammatory conditions. Cells. 2023;12(14):1824. 37508489 10.3390/cells12141824 PMC10377825

