# Supplementary material for: In vitro assays for investigating the immunomodulatory properties of human mesenchymal stromal cells
Source: Stem Cell Res Ther. 2026 Feb 7;17:102. doi: 10.1186/s13287-026-04920-x (PMC12977495; doi:10.1186/s13287-026-04920-x)
Supplement: Supplementary file 1 — Supplementary Material 1. [file 13287_2026_4920_MOESM1_ESM.docx]

Supplementary Table 1 Overview of included immune cells.

| **Cell types** | **References** |
| --- | --- |
| PBMC (54.5%) | (3-5, 7, 13-18, 29, 41, 43-45, 47, 48, 50, 53, 56, 58-61, 64, 66, 68-72, 74, 77-83, 85, 88, 97, 107, 113, 114, 119-121, 126, 127, 130-135, 138, 140-142, 146, 149, 155-158, 164, 166-171, 175-179, 182-188, 190, 192-206, 208, 210, 214, 217, 218, 220, 221, 227, 231-237, 239, 241, 242, 245-250, 256, 257, 262-266, 269-271, 276-279, 282, 284, 286, 288, 289, 294-296, 298, 299, 302, 312, 313, 315, 316, 318-323, 325, 328, 330-334, 336-338, 340) |
| T cells (18.9%) | (9, 42, 44, 45, 49, 60, 62, 64, 73, 77, 78, 82, 84-86, 89, 94, 99, 103, 112, 121, 122, 133, 139, 147, 159-162, 165, 174, 189, 207, 211, 219, 220, 225, 240, 241, 244, 251, 255, 259, 261, 268, 276, 290, 301, 305, 307-310, 314, 317, 319, 324, 326, 327, 329, 341) |
| Macrophages (13.6%) | (11, 13, 14, 46, 58, 67, 78, 90-105, 108-116, 151, 154, 174, 185, 191, 220, 239, 254, 255, 260, 321, 335) |
| Monocytes (3.7%) | (11, 13, 16, 19, 88, 106, 107, 191, 209, 298, 306, 317) |
| NK cells (2.5%) | (40, 117-120, 227, 253, 310) |
| Neutrophils (2.2%) | (87, 90, 97, 185, 213, 215, 258) |
| B cells (1.9%) | (65, 82, 99, 163, 212, 310) |
| Dendritic cells (1.9%) | (178, 220, 230, 255, 321, 339) |
| Full blood (0.6%) | (87, 88) |
| Glial cells (0.3%) | (173) |

Table listing references for included immune cells.

Supplementary Table 2 Overview of the priming reagents used.

| Priming agent | References |
| --- | --- |
| Interferon (IFN)γ | (8, 12, 14-16, 19, 41, 43-45, 57, 58, 60, 62-64, 66, 67, 71, 73, 77, 78, 85, 87, 93, 97, 99, 100, 102, 105, 114, 120, 128, 142, 156, 157, 159, 162, 164, 166, 169-171, 180, 182, 185, 186, 196, 203, 214, 216, 217, 219, 221-223, 226, 227, 229, 234-236, 238, 239, 241, 243, 244, 246-248, 250, 251, 268, 269, 276-279, 282, 284-286, 288-290, 292-299, 301-303, 308, 310, 315, 321, 330, 331, 338, 342, 343) |
| Tumor necrosis factor (TNF)α | (10, 12, 14-16, 19, 41, 44, 45, 55, 57, 58, 60, 62-64, 71, 75, 78, 85, 93, 102, 105, 128, 154, 157, 159, 160, 182, 184-186, 196, 198, 203, 216, 217, 222, 226, 227, 229, 234, 236, 239-241, 244, 247, 262, 269, 277-280, 282-286, 290, 294, 295, 301, 302, 308, 310, 331, 338, 344) |
| Interleukin (IL)1β | (12, 14, 19, 55, 62, 75, 95, 105, 115, 159, 160, 162, 165, 185, 186, 191, 226, 227, 229, 234, 239, 240, 262, 284, 290, 294, 323, 331, 338) |
| Other proinflammatory cytokines | (12, 63, 105, 154, 156, 234, 288, 290, 295, 308, 315, 331, 338) |
| Toll-like receptor agonists | (5, 13, 57, 73, 76, 97, 109, 118, 155, 161, 167, 214, 238, 265, 276, 279, 281, 283, 284, 291, 292, 300) |

Table listing references for each type of priming reagent used.

Supplementary Table 3 Overview of methods used for characterization of primed MSCs.

| Methods used for characterization of primed MSC | References |
| --- | --- |
| PCR | (5, 8, 12, 16, 55, 62, 73, 75, 76, 78, 85, 102, 105, 109, 115, 118, 154, 156-161, 166, 168, 170, 182, 184, 186, 198, 214, 216, 217, 226, 227, 238-241, 243, 244, 265, 276-285, 295, 299, 315, 321, 342, 343) |
| ELISA | (13, 45, 58, 63, 76, 95, 97, 99, 109, 128, 154-159, 165, 166, 168, 184, 196, 217, 222, 226, 227, 239-241, 243, 247, 248, 251, 265, 268, 278, 279, 282-284, 286, 288-294) |
| Sequencing | (10, 19, 55, 66, 100, 120, 155, 161, 171, 196, 198, 217, 222, 236, 251, 265, 269, 276, 279, 281, 283, 285, 290, 293, 295-297) |
| Western blot | (15, 16, 62, 73, 78, 102, 115, 128, 157, 164, 171, 182, 186, 226, 244, 292, 298, 299) |
| Luminex | (5, 55, 57, 58, 164, 167, 180, 223, 226, 229, 236, 246, 276, 279, 289, 299-301) |
| Flow cytometry | (12, 19, 66, 71, 77, 142, 154, 157, 159, 180, 182, 184, 221, 234, 238-240, 247, 251, 282, 290, 302, 303) |
| Mass spectrometry | (58, 87, 100, 109, 246, 250, 277) |
| Colorimetric assay | (85, 162, 239, 241, 244, 248, 292) |
| Microscopy | (102, 128, 288) |
| NMR spectroscopy | (246, 250) |

References for methods used to investigate the effect of priming on the immunomodulatory properties of MSCs.
